# Multiscale mechanistic modeling for the rational design of novel dual-target candidates against acetylcholinesterase and NADPH oxidase: an advanced computational study

**DOI:** 10.3389/fchem.2026.1917677

**Published:** 2026-08-21

**Authors:** Mohamed El Fadili, Mohammed Er-rajy, Somdutt Mujwar, Mourad Aloui, Abdelouahid Samadi, Samir Chtita, Menana Elhallaoui

**Affiliations:** 1 LIMAS Laboratory, Faculty of Sciences Dhar El Mahraz, Sidi Mohamed Ben Abdellah University, Fez, Morocco; 2 Chitkara College of Pharmacy, Chitkara University, Rajpura, Punjab, India; 3 Department of Chemistry, College of Science, United Arab Emirates University, Al Ain, United Arab Emirates; 4 Laboratory of Analytical and Molecular Chemistry, Faculty of Sciences Ben M’Sik, Hassan II University of Casablanca, Casablanca, Morocco

**Keywords:** 3D-QSAR, Alzheimer’s disease, cholinesterase, molecular dynamics, oxidative stress

## Abstract

**Introduction:**

Alzheimer’s disease is a complex neurodegenerative illness strongly associated with oxidative stress, which requires immediate intervention for therapeutic agents with potent antioxidant properties.

**Methods:**

In this work, novel derivatives based on benzofuran and pyrazole scaffolds were designed and assessed for their antioxidant potential and acetylcholinesterase inhibitory activity. The identification of key molecular characteristics that are important for the ability of a compound to scavenge DPPH radicals and AChE cholinesterase and has been accomplished through SAR, then both CoMFA and CoMSIA 3D-QSAR modelling have been used to design fifteen new compounds (D1-D15) which inhibit both DPPH and AChE to a significantly greater extent than the parent compound. Density functional theory calculations at the B3LYP/6-31G (d,p) level revealed that the three most promising candidates (M12, D8, and D9) have quite high electronic and molecular stability.

**Results and Discussion:**

The results of pharmacokinetic tests, molecular docking, and 100 ns molecular dynamics experiments confirm that these examined compounds interact favorably with both human acetylcholinesterase and NADPH oxidase enzymes with excellent thermodynamic stability. These results provide further evidence for the potential of the designed compounds to behave as multi-target ligands with AChE inhibition and antioxidant properties that would be effective in the treatment of the complex Alzheimer’s disease.

## 1 Introduction

Alzheimer’s disease (AD) is an ever-worsening neurological disorder that affects overall function (memory, ability to learn new material, and ability to think) and can ultimately lead to losing independence or a good quality of life (El fadili et al., 2023a). The etiology of AD is multifactorial, including inhibitors of acetylcholinesterase (AChE), NMDA receptor antagonists, and several other factors such as amyloid-β plaque accumulation, hyperphosphorylated tau protein formation, synaptic dysfunction (failure), and oxidative stress. As such, creating effective therapies to treat AD is a difficult task. Currently, available medications such as AChE inhibitors and memantine provide only temporary relief from the symptoms of AD; additionally, they have numerous adverse side effects and complications when taken for long periods of time, such as neurologic, cardiovascular, and gastrointestinal complications, thus limiting their ability to be clinically effective. (El Fadili et al., 2025). There is an inherent need to discover new therapeutic approaches that deal with the complexity of AD-related issues (El Khatabi et al., 2022).

Oxidative stress is one of the primary mechanisms involved in the pathology of AD; excessive amounts of reactive oxygen species (ROS) cause neuronal cell death, mitochondrial dysfunction, and synaptic loss. NADPH oxidase complex has been identified as a significant source of ROS in the CNS, and more recently, it has emerged as a potential therapeutic target for the treatment of neurodegenerative diseases (Block, 2008). Structural data, including the crystallographic model of human NADPH oxidase (PDB ID: 2CDU), offer an opportunity to design antioxidant agents capable of attenuating oxidative damage and slowing disease progression (Lountos et al., 2006). Pyrazole- and benzofuran-based derivatives recently synthesized by Elkotamy et al., include beneficial characteristics in terms of cholinesterase inhibition and antioxidative effectiveness. As oxidative stress plays an important role in AD, while NADPH oxidases contribute considerably to ROS generation, the multifunctional scaffolds presented in the present work were thought to be of interest for testing their ability to modulate oxidative stress at the enzymatic source. Thus, the current study aims to broaden the pharmacological profile of such compounds using molecular docking studies with NADPH, followed by molecular dynamics modeling and complementary *in silico* characterization (Elkotamy et al., 2025).

In recent years, the concept of multifunctional ligands has gained momentum as a strategy for AD therapy. Compounds that combine cholinesterase inhibition with antioxidant activity may provide synergistic neuroprotective effects, simultaneously modulate cholinergic transmission, and reduce oxidative stress. Advances in computational drug discovery now enable a rational exploration of such multitarget agents (Elkotamy et al., 2025).

Previous computational investigations targeting AD have principally focused on single molecular targets, like AChE to restore cholinergic conduction (Mehta et al., 2012; Sharma, 2019; Xu et al., 2021; Zuliani et al., 2024), the NMDA receptor to regulate glutamatergic hyperactivation (Karimi Tari et al., 2024; Liu et al., 2019; Olivares et al., 2012; Reisberg et al., 2003), or β-amyloid assembly to reduce plaque formation (Hampel et al., 2021; Høilund-Carlsen et al., 2024; Zhang et al., 2023). Although these investigations have provided exact insights into individual pathological mechanisms, their limited scope fails to reflect the multidimensional nature of the disease. The current study expands upon the previously mentioned research topic by creating a multi-target method, which contains the oxidative stress-causing nature of NADPH oxidase, and uses both antioxidant and cholinesterase inhibition. This multi-target approach will allow us to provide a wider perspective of potential treatment options, as well as an improved comprehensive approach to developing multifunctional agents for the treatment of AD. Structure-based approaches, including molecular docking and molecular dynamics (MD) simulations, are powerful methods for understanding the binding affinity, stability, and conformational adaptability of compounds to their target proteins (Aloui et al., 2025; El fadili et al., 2023d). Additionally, three-dimensional quantitative structure-activity relationship (3D-QSAR) models (Er-rajy et al., 2024) and density functional theory (DFT) calculations (El fadili et al., 2023c), will provide insight into the identification of important pharmacophore characteristics, as well as how the electronic properties of the designed multi-functional agent can be improved, guiding the design of potent and selective molecules (El fadili et al., 2022a). In addition, a drug-likeness assessment based on Lipinski’s and other rules guarantees that the potential scaffolds will have good pharmacokinetic properties (Benkhaira et al., 2025; Ed-Dahmani et al., 2024). The present study has implemented an integrated *in silico* method for identifying and characterizing novel multifunctional ligands, which would target the NADPH oxidase receptor (Ed-Dahmani et al., 2025; Nouioura et al., 2024b). To achieve this, we will be performing docking studies on the structure of 2CDU.pdb and carrying out extensive MD simulations to elucidate the molecular determinants responsible for the dual activity of these new compounds. These compounds are expected to be very good candidates for development as multitarget therapeutics in the treatment of AD, as they possess both antioxidant activity and cholinesterase inhibition properties (Ed-Dahmani et al., 2024).

## 2 Materials and methods

### 2.1 Experimental database

To pursue the development of two families of novel compounds to target the multiple pathologies involved with AD, we have developed benzofuran-containing donepezil derivatives (M1-M9) and pyrazole-containing analogues (M10-M18), which are shown in Table 1. The rationale for designing these compounds was to create a dual-target (cholinergic and oxidative stress) molecule with their primary activity being cholinesterase inhibition while providing enhanced antioxidant properties (Elkotamy et al., 2025). The rationale for screening these molecules was due to the complex and multifactorial nature of the pathophysiology of AD, along with overlapping mechanisms (cholinergic dysregulation and oxidative stress) and undesirable side effects associated with single-target drugs. Therefore, it is a logical step to develop multifunctional compounds that would inhibit both oxidative stress and AChE. The concept of dual-action function is supported by previous studies showing that these hybrid derivatives have been shown to exert both neuroprotective and antioxidant actions *in vitro* and *in vivo* 6. This rationale underlies our current study, which will utilize an integrated *in silico* approach to identify the most promising multifunctional compounds through the use of 3D-QSAR, DFT calculations, ADMET evaluation, molecular docking, and molecular dynamics.

**TABLE 1:** AChE and DPPH IC_50_ activities for novel pyrazole and benzofuran-based derivatives.

N°	Structure	R_1_	R_2_	R_3_	AChE IC_50_ (µg/mL)	DDPH IC_50_ (µg/mL)
M1	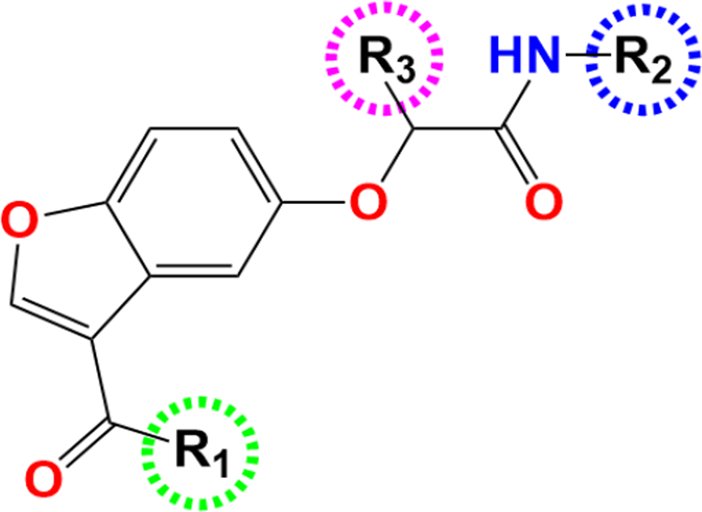	Ph	Ph	H	0.77	587
M2		Ph	4-Me-C_6_H_4_	H	8.26	262
M3		Ph	4-Cl-C_6_H_4_	H	16.46	132
M4		4-Me-C_6_H_4_	Ph	H	1.24	363
M5		4-Me-C_6_H_4_	4-Me-C_6_H_4_	H	23.67	240
M6		4-Me-C_6_H_4_	4-Cl-C_6_H_4_	H	21.82	732
M7		4-Cl-C_6_H_4_	Ph	H	0.39	153
M8		4-Cl-C_6_H_4_	4-Me-C_6_H_4_	H	5.01	375
M9		4-Cl-C_6_H_4_	4-Cl-C_6_H_4_	H	7.96	630
M10	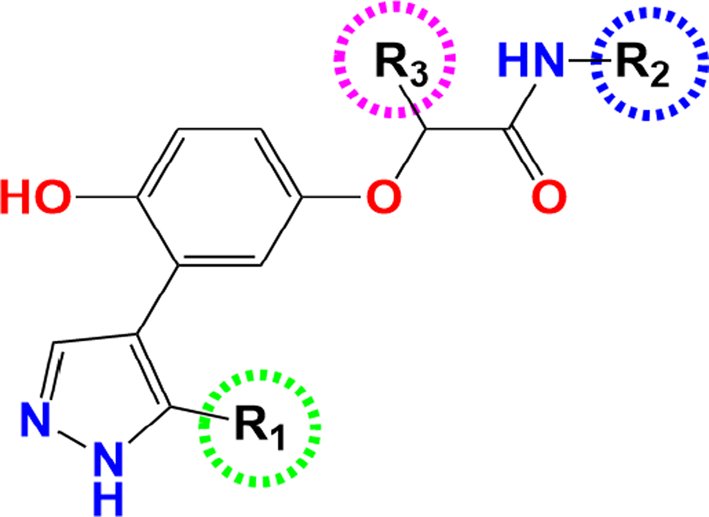	Ph	Ph	H	19.35	3.07
M11		Ph	4-Me-C_6_H_4_	H	11.7	9.93
M12		Ph	4-Cl-C_6_H_4_	H	17	2.59
M13		4-Me-C_6_H_4_	Ph	H	16.3	3.24
M14		4-Me-C_6_H_4_	4-Me-C_6_H_4_	H	1.97	4.26
M15		4-Me-C_6_H_4_	4-Cl-C_6_H_4_	H	1.24	3.15
M16		4-Cl-C_6_H_4_	Ph	H	5.75	5.87
M17		4-Cl-C_6_H_4_	4-Me-C_6_H_4_	H	18.81	4.94
M18		4-Cl-C_6_H_4_	4-Cl-C_6_H_4_	H	54.01	3.18

### 2.2 3D-QSAR study

**FIGURE 1:**
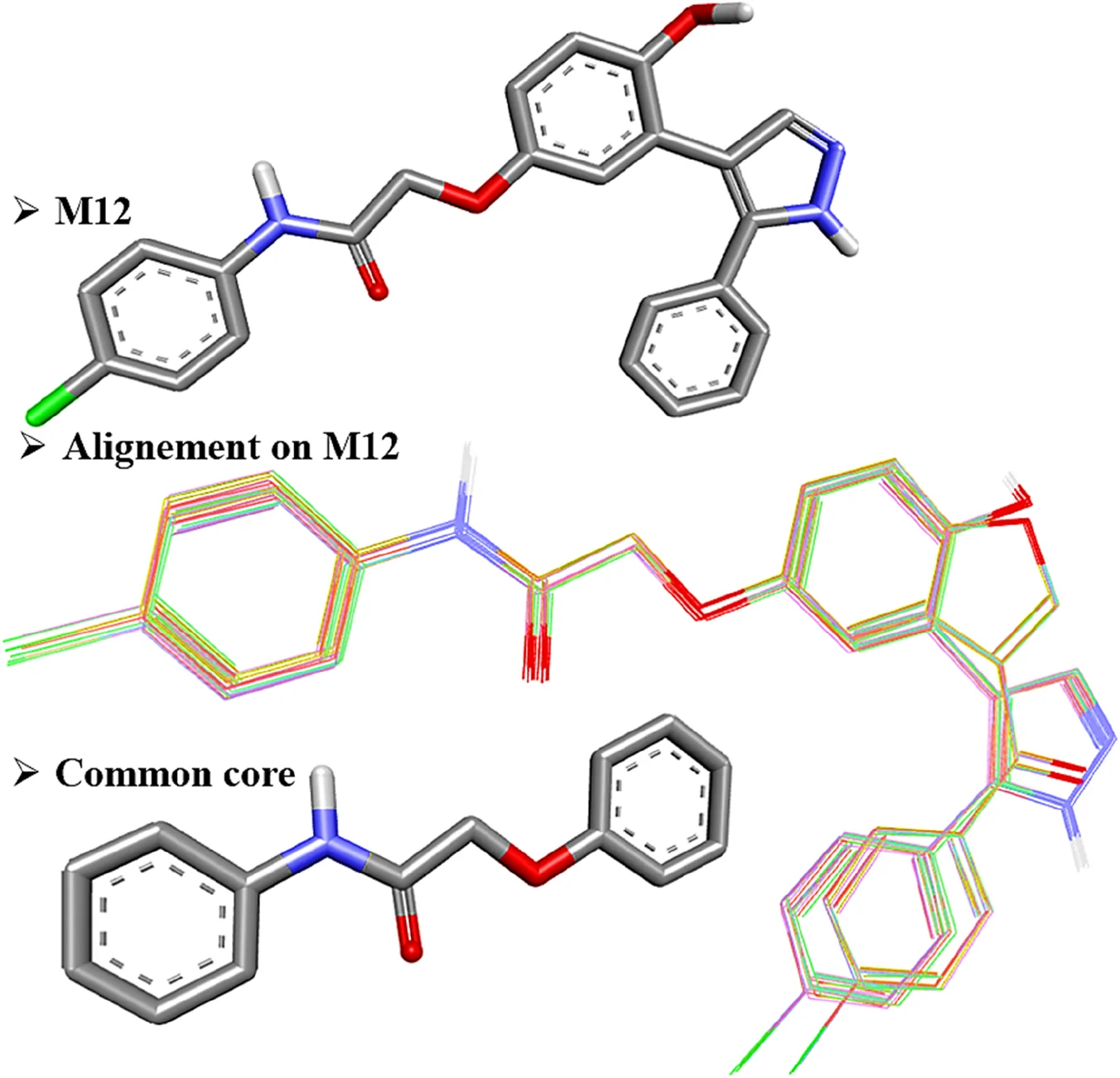
2D structures of the synthesized compound with the most antioxidant efficiency (M12), the superposition result for both pyrazole- and benzofuran-based derivatives, and the resulting common core.

3D-QSAR analyses of newly designed pyrazole and benzofuran derivatives for anticholinesterase and antioxidant potential were performed with SYBYL-X 2.0 using the partial least squares (PLS) method to generate a classical CoMFA model and several CoMSIA models (Er-rajy et al., 2024). The robustness of the analyses was ensured with randomization of the experimental data into two groups of compounds: a training set that included 70% of the compounds and a test set that included the remaining 30%. To ensure robustness, the experimental data were randomized and divided into two groups: a training set covering 70% of the compounds and a test set comprising the remaining 30%. The CoMFA approach considered steric and electrostatic fields, while CoMSIA additionally accounted for hydrophobic properties and hydrogen-bond donor and acceptor interactions. The predictive ability of the models was externally validated using a test set of compounds M2*, M10*, M14*, M15*, and M16*, whereas the remaining molecules were employed to construct the models. Among these, M12, a benzofuran derivative, exhibited the strongest antioxidant effect. Before model development, molecular geometries were optimized by energy minimization using the Tripos force field, with Gasteiger-Hückel charges, a Powell-gradient convergence criterion of 0.001 kcal (mol·Å), and a maximum of 10,000 iterations. Structural alignment highlighted 2-phenoxy-N-phenylacetamide as the common pharmacophoric scaffold shared by both chemical series, as presented in Figure 1.

### 2.3 Drug-likeness and ADMET *in silico* evaluation

A major objective in contemporary drug discovery is to identify molecules that combine therapeutic efficacy with an acceptable safety profile (Ajala et al., 2023; Jeddi et al., 2023), while reducing the time and cost associated with experimental trials. In this context, computer-aided drug design (CADD) provides valuable predictive insights. At this stage of the study, the pharmacokinetic characteristics of the proposed compounds, including absorption, distribution, metabolism, excretion, and toxicity (ADMET) were estimated through the SwissADME and pkCSM web-based platforms (Bouzammit et al., 2024a; Nouioura et al., 2024a).

### 2.4 DFT calculation studies

DFT is a key computational method in quantum chemistry used to study molecular structures and electronic properties. In this research, DFT was used to optimize the geometries of M12, D8, and D9 derivatives. The process involved the use of the B3LYP hybrid function with the 6-31G (d,p) basis set for initial optimization (Ez-zoubi et al., 2025), followed by further refinement with the same basis set for increased accuracy. All calculations were performed using Gaussian 09W, providing reliable results based on well-established quantum mechanical principles. After optimization, the structures were visualized and analyzed using GaussView-6 in order to assess the molecular conformation and electronic distribution of these compounds. This work will provide important data regarding the electronic characteristics and stability of the compounds, which will be a basis for both future experimental studies and further theoretical research (Nimr Ajeel et al., 2017).

### 2.5 Docking simulations

Molecular docking simulations were performed using Discovery Studio 2021 and AutoDock 4.2 to assess the binding of the selected compounds to human NAD(P)H oxidase (PDB ID 2CDU) and AChE cholinesterase (PDB ID od 4EY7). The examined compounds (M12, D8 and D9) were well evaluated. Before docking, the protein structures were prepared by removing all water molecules and co-crystallized ligands (Bouzammit et al., 2025; Rani et al., 2023) and adding Gasteiger charges to give an accurate representation of electrostatic interactions (Bouzammit et al., 2024b; El fadili et al., 2023b). The docking grid boxes were centered on the target proteins at Cartesian coordinates of x = 10.20, y = 0.66, z = 6.15 for the 2CDU protein, and x = −2.95, y = −40.107, z = 30.729 for the 4EY7 protein with the same grid spacing of 0.375 Å. After docking, Discovery Studio 2021 was employed to visualize and analyze the resulting ligand-protein interactions in both 3D and 2D representations (Abechi et al., 2024; El fadili et al., 2022b).

### 2.6 Molecular dynamics simulations

Dynamic behavior of the ligand-protein complexes was evaluated through 100 ns MD simulations using the Desmond module of Schrödinger Software. MD Simulations were performed using the OPLS4 force field with an explicit TIP3P water model. M12-NADPH oxidase (PDB ID: 2CDU), D8-NADPH oxidase (PDB ID: 2CDU), and D9-AChE (PDB ID: 4EY7) complexes were solvated and neutralized; complexes were equilibrated using NVT and NPT ensembles at 300 K and 1 bar (El Fadili et al., 2026). Structural stability, flexibility, and binding integrity were analyzed using RMSD, RMSF, and hydrogen bond interactions (Er-rajy et al., 2023; Zarougui et al., 2024). Analyses were performed to provide detailed insight into ligand conformational dynamics within the active sites of both AChE and NADPH oxidase enzymes, providing further support for these compounds as potential therapeutic agents for AD through dual targeting of oxidative stress and cholinergic dysfunction.

## 3 Results and discussions

### 3.1 3D-QSAR study

Multiple three-dimensional quantitative structure-activity Relationship models have been constructed with the use of PLS regression analyses to predict the DPPH radical scavenging and AChE IC_50_ inhibitory activities of newly synthesized pyrazole- and benzofuran-derived substances (Table 2). Various descriptor types were tested, including steric (S), electrostatic (E), hydrophobic (H), hydrogen bond donor (D), and hydrogen bond acceptor (A) descriptor types using the methods of CoMFA and CoMSIA. A CoMFA analysis includes only steric and electrostatic types of field descriptors, whereas a CoMSIA analysis incorporates information corresponding to all five descriptor types (S, E, H, D, and A). To establish the reliability and predictive capacity of the models developed, several statistical metrics were used. The Q2cv value for a reliable model derived via leave-one-out (LOO) validation should exceed 0.6 (El fadili et al., 2024), and the non-cross-validated R2 value of a reliable model must exceed 0.7. An acceptable model requires (1) that the Fisher ratio (F) exceed its theoretical limit; (2) that it has a low standard error of estimate (SEE); and (3) that there are an appropriate number of optimal components identified within the model. External validation of predictive capacity was also performed; the R2 value for the test set must exceed the 0.6 threshold.

**TABLE 2:** Statistical outcomes of 3D-QSAR models across different molecular field combinations for predicting both DPPH and AChE IC_50_ inhibitory activities.

CoMFA and CoMSIA models of both DPPH and AChE		Fractions of molecular interactions fields					*Q* ^*2*^_*cv*_	*R* ^*2*^	*SEE*	*F*	*ONC*	*R* ^*2*^ *pr*
		(S)	(E)	(H)	(D)	(A)						
COMFA	DPPH model	0.266	0.734	-	-	-	0.868	0.919	0.298	124.026	1	0.912
	AChE model	0.542	0.458	-	-	-	0.797	0.949	0.169	55.609	3	0.888
CoMSIA SEA	DPPH model	0.006	0.409	-	-	0.585	0.908	0.934	0.267	156.741	1	0.983
	AChE model	0.079	0.815	-	-	0.105	0.793	0.988	0.121	42.433	8	0.732
CoMSIA SEH	DPPH model	0.012	0.820	0.167	-	-	0.900	0.933	0.270	152.969	1	0.981
	AChE model	0.041	0.579	0.380	-	-	0.811	0.975	0.158	28.016	7	0.930
CoMSIA SED	DPPH model	0.005	0.346	-	0.649	-	0.908	0.935	0.267	157.108	1	0.983
	AChE model	0.080	0.828	-	0.092	-	0.775	0.988	0.121	42.581	8	0.732
CoMSIA SHD	DPPH model	0.007	-	0.097	0.896	-	0.909	0.935	0.266	158.040	1	0.979
	AChE model	0.060	-	0.558	0.382	-	0.762	0.975	0.158	27.916	7	0.952
CoMSIA SHA	DPPH model	0.009	-	0.124	-	0.867	0.908	0.935	0.266	157.720	1	0.979
	AChE model	0.059	-	0.533	-	0.408	0.822	0.975	0.159	27.424	7	0.946
CoMSIA SDA	DPPH model	0.005	-	-	0.565	0.430	0.911	0.935	0.266	158.746	1	0.981
	AChE model	0.256	-	-	0.275	0.468	0.788	0.938	0.210	21.263	5	0.863
CoMSIA EHA	DPPH model	-	0.380	0.077	-	0.543	0.907	0.935	0.267	157.135	1	0.982
	AChE model	-	0.485	0.378	-	0.138	0.818	0.988	0.122	41.828	8	0.964
CoMSIA EHD	DPPH model	-	0.325	0.066	0.609	-	0.908	0.935	0.267	157.413	1	0.982
	AChE model	-	0.496	0.388	0.116	-	0.802	0.988	0.122	41.896	8	0.965
CoMSIA EDA	DPPH model	-	0.232	-	0.436	0.332	0.909	0.935	0.266	158.051	1	0.982
	AChE model	-	0.865	-	0.055	0.081	0.799	0.988	0.108	60.261	7	0.734
CoMSIA HDA	DPPH model	-	-	0.058	0.535	0.407	0.910	0.935	0.266	158.820	1	0.980
	AChE model	-	-	0.568	0.167	0.265	0.787	0.974	0.161	26.952	7	0.876
CoMSIA EHDA	DPPH model	-	0.222	0.045	0.416	0.317	0.909	0.935	0.266	157.982	1	0.981
	AChE model	-	0.454	0.368	0.074	0.104	0.810	0.988	0.122	41.555	8	0.964
CoMSIA SHDA	DPPH model	0.004	-	0.058	0.532	0.406	0.910	0.935	0.266	158.537	1	0.980
	AChE model	0.057	-	0.489	0.174	0.280	0.800	0.974	0.160	27.227	7	0.945
CoMSIA SEDA	DPPH model	0.003	0.231	-	0.434	0.331	0.909	0.935	0.266	157.814	1	0.982
	AChE model	0.034	0.644	-	0.143	0.178	0.784	0.988	0.121	41.994	8	0.736
CoMSIA SEHA	DPPH model	0.006	0.377	0.077	-	0.540	0.907	0.934	0.267	156.697	1	0.982
	AChE model	0.039	0.466	0.367	-	0.128	0.821	0.988	0.124	40.236	8	0.971
CoMSIA SEHD	DPPH model	0.005	0.323	0.066	0.606	-	0.907	0.935	0.267	157.049	1	0.982
	AChE model	0.028	0.433	0.293	0.245	-	0.806	0.973	0.163	26.060	7	0.896
CoMSIA SEHDA	DPPH model	0.003	0.221	0.045	0.415	0.316	0.908	0.935	0.266	157.747	1	0.981
	AChE model	0.038	0.435	0.357	0.072	0.097	0.825	0.988	0.125	39.863	8	0.972
Total fractions of 1							External and internal validations					

Abbreviations: (S): Steric fields, (E): Electrostatic fields, (H): Hydrophobic fields, (D): Donors of hydrogen bond, (A): Acceptors of hydrogen bond, *Q* ^*2*^_*cv*_: the cross-validation determination coefficient, *R* ^2^: the non-cross-validation determination coefficient, ***SEE***: the stann v v b v v *ùdard estimation error, ***F***: the Fischer test value, ***ONC***: the optimum number of components, and *R* ^2^ pr: the external validation determination coefficient.

A total of two CoMFA models and sixteen CoMSIA variants were constructed for both the DPPH and AChE datasets using the training sets. Among them, the best-performing models for the DPPH dataset were CoMFA-2 (R^2^ = 0.919, Q^2^cv = 0.868, R^2^pred = 0.912) and CoMSIA/SDA (R^2^ = 0.935, Q^2^cv = 0.911, R^2^pred = 0.981). For the AChE dataset, the best predictive performance was achieved by CoMFA-1 (R^2^ = 0.949, Q^2^cv = 0.797, R^2^pred = 0.888) and CoMSIA/SEHDA (R^2^ = 0.988, Q^2^cv = 0.825, R^2^pred = 0.972).

**FIGURE 2:**
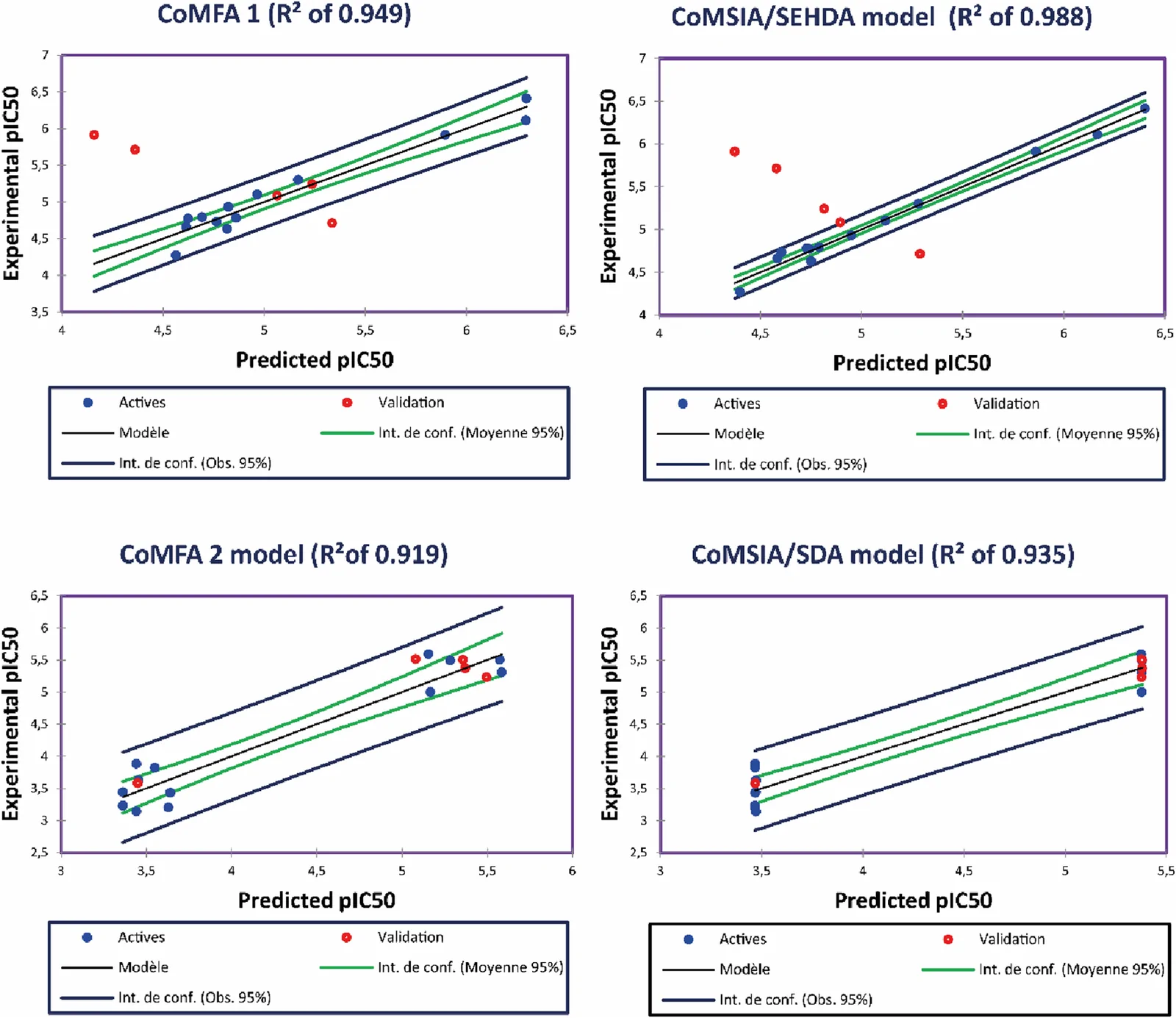
Correlation between the experimental and predicted AChE and DPPH-antagonizing activities using validated CoMFA and CoMSIA models, respectively. CoMFA 1 and CoMSIA/SEHDA models were previously reported by (El Fadili et al., 2025) and are adapted under the Creative Commons Attribution License (CC BY).

The values generated from the selected models were strongly consistent in both internal and external validation, thereby confirming that steric, electrostatic, hydrophobic, and hydrogen-bond donor/acceptor interaction fields are critical in modulating AChE and DPPH inhibitory activities. The predicted IC_50_ values for both test and training compounds of the AChE and DPPH datasets are presented in Table 3, while the correlations between experimental and predicted activities are illustrated in Figure 2, where test and training-set molecules are represented in red and blue, respectively.

**TABLE 3:** Prediction of AChE and DPPH activities using validated CoMFA and CoMSIA models.

N°	AChE IC_50_ (µg/mL)	DDPH IC_50_ (µg/mL)	AChE pIC_50_	DPPH pIC_50_	Predicted pIC_50_ using CoMFA-1 model	Predicted pIC_50_ using CoMFA-2 model	Predicted pIC_50_ using CoMSIA- SEHDA model	Predicted pIC_50_ using CoMSIA- SDA model
M1	0.77	587	6.11	3.23	6.295	3.362	6.166	3.468
M3	16.46	132	4.78	3.88	4.862	3.442	4.731	3.469
M4	1.24	363	5.91	3.44	5.895	3.363	5.862	3.471
M5	23.67	240	4.63	3.62	4.818	3.455	4.751	3.473
M6	21.82	732	4.66	3.14	4.615	3.442	4.586	3.472
M7	0.39	153	6.41	3.82	6.297	3.549	6.401	3.469
M8	5.01	375	5.3	3.43	5.169	3.641	5.283	3.47
M9	7.96	630	5.1	3.2	4.966	3.629	5.119	3.469
M11	11.7	9.93	4.93	5	4.824	5.167	4.950	5.378
M12	17	2.59	4.77	5.59	4.624	5.156	4.744	5.376
M13	16.3	3.24	4.79	5.49	4.694	5.284	4.791	5.38
M17	18.81	4.94	4.73	5.31	4.765	5.585	4.605	5.378
M18	54.01	3.18	4.27	5.5	4.565	5.574	4.400	5.377
M2*	8.26	262	5.08	3.58	5.065	3.45	4.896	3.47
M10*	19.35	3.07	4.71	5.51	5.336	5.08	5.290	5.377
M14*	1.97	4.26	5.71	5.37	4.362	5.372	4.580	5.381
M15*	1.24	3.15	5.91	5.5	4.161	5.359	4.374	5.38
M16*	5.75	5.87	5.24	5.23	5.237	5.496	4.817	5.377

* Refers to test set molecules.

Even though a small amount of data was utilized to create the QSAR models (n = 18), it largely reflects the low availability of current pyrazole- and benzofuran-based compounds, which have been previously synthesized and tested for DPPH radical scavenging and AChE inhibition. Despite good internal and external validation results, the results obtained must be treated with caution regarding the prediction potency of the models developed. Future work will involve much greater datasets with diverse structures.

### 3.2 A graphical analysis of validated 3D-QSAR models

**FIGURE 3:**
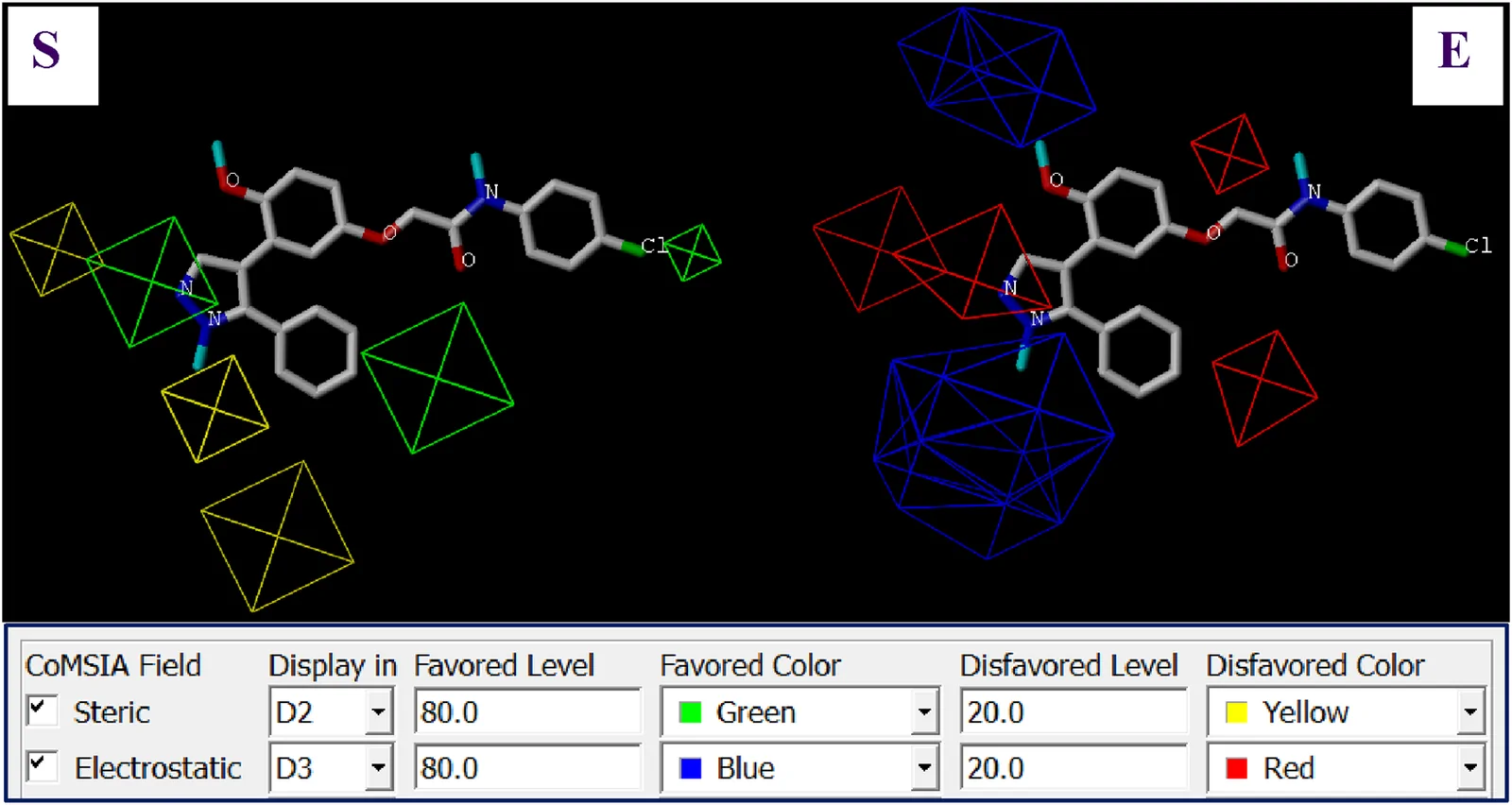
Steric (S) and electrostatic (E) molecular fields implicated in the CoMFA-2 model for M12.

**FIGURE 4:**
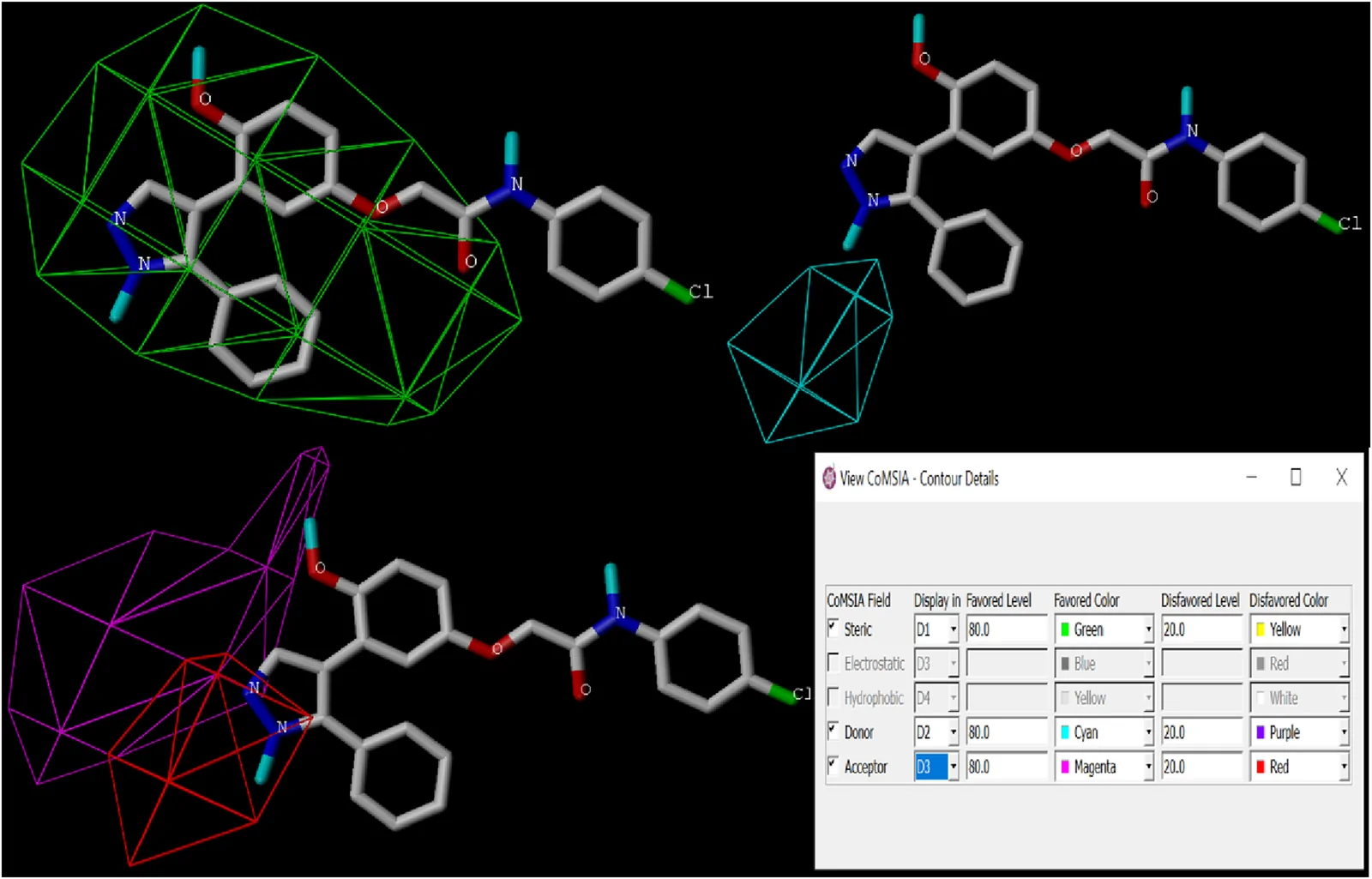
Steric (S), Donor (D) and Acceptor (A) molecular interaction fields predicted by CoMSIA SDA model for M12.

**FIGURE 5:**
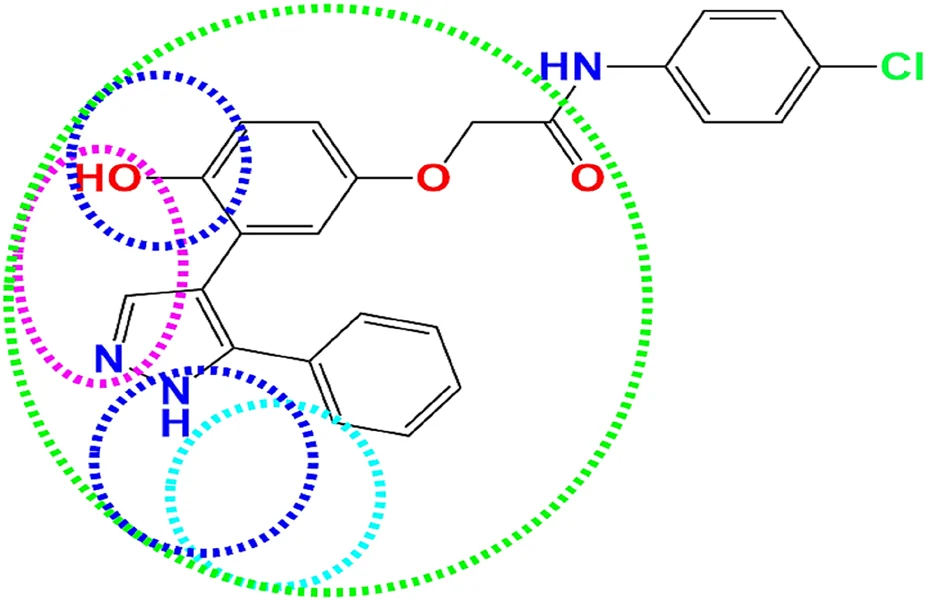
Favorable regions of various molecular fields derived from 3D-QSAR models: Steric (in green), Electrostatic (in blue), Acceptor (in magenta), and Donor of hydrogen bonds (in cyan).

Three-dimensional contour maps generated from the CoMFA and CoMSIA models were employed to pinpoint critical regions affecting steric, electrostatic, hydrophobic, and hydrogen-bond donor acceptor properties, which contribute to the antioxidant activity of the pyrazole and benzofuran derivatives. The results indicate that CoMFA and CoMSIA SDA models were approved as the best 3D-QSAR models, in which steric fields colored in green are favored by a large region (almost the entire capsule) of the M12 molecule as a highly antioxidant-active compound, while electrostatic molecular fields marked in blue are located around the first aromatic ring (Ar1) linked to pyrazole core and the medium aromatic ring linked to alcohol function, as shown in Figure 3. Close to the same region of the template molecule, Steric, Donor, and Acceptor fields located in the vicinity of the pyrazole nucleus promote the antioxidant activity of novel pyrazole- and benzofuran-based derivatives (Figure 4). The mapped favorable interaction areas, including steric and electrostatic, donor and acceptor fields from CoMFA and CoMSIA SDA models, establish a three-dimensional structure-activity relationship (SAR) between DPPH inhibitory activity potential and the most active derivative (M12), as depicted in Figure 5. Based on these structural insights, the development of the first series of pyrazole derivatives (D1–D8) was done according to structural substitutions obtained from the most effective synthesized compound against DPPH (M12). The next series of benzofuran derivatives (D9–D15) was obtained by applying the computed structure-activity relationship (SAR) of the most active synthetic AChE inhibitor (M7). The predicted pIC50 values of all the developed compounds for the DPPH radical scavenging and AChE inhibitory activity are given in Table 4.

**TABLE 4:** Predicted both DPPH and AChE pIC_50_ values of novel compounds designed using validated CoMFA and CoMSIA models.

I. DPPH predictions						
N°	Structure	R_1_	R_2_	R_3_	Predicted pIC_50_ using CoMFA-2 model	Predicted pIC_50_ using CoMSIA-SDA model
M12	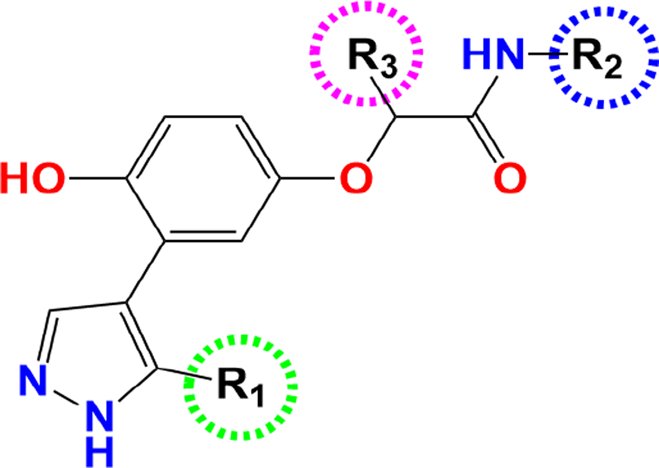	Ph	4-Cl-C_6_H_4_	H	5.156	5.376
D1		C_6_H_4_	C_6_H_4_	NH_2_	3.261	3.528
D2		C_6_H_4_	4-Cl-C_6_H_4_	NH_2_	3.446	3.633
D3		4-Cl-C_6_H_4_	4-Cl-C_6_H_4_	NH_2_	3.641	3.634
D4		4-Br-C_6_H_4_	C_6_H_4_	CH_3_	3.647	3.431
D5		5-Cl-C_6_H_4_	C_6_H_4_	CH_3_	3.477	3.431
D6		5-Cl-C_6_H_4_	5-Cl-C_6_H_4_	CH_3_	3.675	3.422
D7		5-Cl-C_6_H_4_	5-Cl-C_6_H_4_	OH	3.661	3.525
D8		5-Cl-C_6_H_4_	5-Cl-C_6_H_4_	NH_2_	3.729	3.523

II. AChE predictions						
N°	Structure	R_1_	R_2_	R_3_	Predicted pIC_50_ using CoMFA-1 model	Predicted pIC_50_ using CoMSIA- SEHDA model
M7	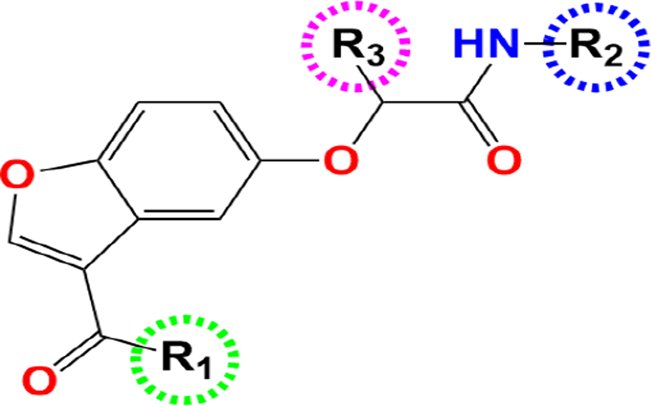	4-Cl-C_6_H_4_	C_6_H_4_	H	6.297	6.401
D9		C_6_H_4_	C_6_H_4_	NH_2_	6.132	6.007
D10		C_6_H_4_	4-Cl-C_6_H_4_	NH_2_	4.735	4.735
D11		4-Cl-C_6_H_4_	4-Cl-C_6_H_4_	NH_2_	4.841	5.122
D12		4-Br-C_6_H_4_	C_6_H_4_	CH_3_	6.019	5.975
D13		5-Cl-C_6_H_4_	C_6_H_4_	CH_3_	6.109	4.021
D14		5-Cl-C_6_H_4_	5-Cl-C_6_H_4_	CH_3_	5.819	3.966
D15		5-Cl-C_6_H_4_	5-Cl-C_6_H_4_	OH	5.901	4.396

### 3.3 Pharmacokinetics profiling

An *in silico* pharmacokinetic evaluation was carried out for the most active derivative (M12) and the newly designed compounds (D8 and D9), covering ADMET parameters. The analysis revealed that M12, although showing strong activity against NADPH oxidase, presented potential safety concerns, as it tested positive in the AMES mutagenicity model and was predicted to cause hepatotoxic effects, which may be linked to genetic alterations and an increased likelihood of rodent carcinogenicity. D8 was developed using the M12 scaffold, which had the best DPPH activity, while D9 utilized C7, the best AChE inhibitor, for better pharmacological parameters and biological activity. The design of D8 and D9 revealed that both compounds exhibited favorable predicted pharmacokinetic properties and increased safety profiles compared to M12, given that they are predicted to be non-mutagenic (AMES negative), non-hepatotoxic, and non-skin sensitizing.

Their bioavailability was nearly identical, with D8 and D9 showing excellent gastrointestinal absorption (90%+) and CNS penetration via BBB passage. Nevertheless, it should be emphasized that both compounds were predicted to inhibit cytochrome P450 isoforms CYP1A2, CYP2C9, CYP2C19, and CYP3A4 (Table 5), which means that they may exhibit the risk of CYP-mediated drug-drug interactions that should be validated in experimental studies.

**TABLE 5:** ADMET pharmacokinetic features of M12, D8, and D9.

Models	ADME and Toxicity properties													
	A	D		M							E	T		
	Intestinal absorption (human)	BBB permeability	CNS permeability	CYP450							Total clearance	AMES toxicity	Hepatotoxicity	Skin sensitization
				Substrate		Inhibitor								
				2D6	3A4	1A2	2C19	2C9	2D6	3A4				
Unity	(%Absorbed)	(Log BB)	(Log PS)	(Yes NO)							(Log mL min^-1^kg^-1^)	(Yes no)		
Predicted values														
M12	85.1	−1.276	−2.177	No	Yes	Yes	Yes	Yes	Yes	Yes	0.097	**Yes**	**Yes**	No
D8	99.922	−0.817	−2.009	No	Yes	Yes	Yes	Yes	No	Yes	0.654	No	No	No
D9	97.525	−0.467	−2.237	No	Yes	Yes	Yes	Yes	No	Yes	0.783	No	No	No

Abbreviations: A: Absorption, D: Distribution, M: Metabolism, E: Excretion, T: Toxicity.

Drug-likeness was further explored in terms of physicochemical properties, using Lipinski’s Rule of Five in conjunction with complementary filters provided by Egan, Veber, Ghose, and Muegge (Table 6). These studies demonstrated that M12, D8, and D9 met the drug-likeness criteria with a molecular weight <500 g/mol, Log P < 5, TPSA<140 A^2^, hydrogen bond acceptors (HBA) ≤10, and hydrogen bond donors (HBD) ≤5, without violating any of the applied drug-likeness rules.

**TABLE 6:** Prediction of physicochemical properties of M12, D8, and D9.

​	TPSA	MW	LogP	n-HBA	n-HBD	Violations number				
						Lipinski	Veber	Egan	Muegge	Ghose
Rule	<140 A^2^	<500 D.a	≤5	<10	<5	≤2	≤2	≤2	≤2	≤2
M12	87.24	419.86	2.47	4	3	Yes	Yes	Yes	Yes	Yes
D8	94.56	455.29	3.59	5	2	Yes	Yes	Yes	Yes	Yes
D9	94.56	386.40	2.53	5	2	Yes	Yes	Yes	Yes	Yes

Abbreviations: TPSA: Topological polar surface area, MW: Molecular weight, LogP: Lipophilicity (octanol/water partition coefficient), n-HBD: number of hydrogen-bond Acceptor, n-HBD: number of hydrogen-bond donors.

### 3.4 Frontier molecular orbital analysis

Frontier Molecular Orbitals (FMOs) analysis, which refers specifically to assessing the Highest Occupied Molecular Orbital (HOMO) and Lowest Unoccupied Molecular Orbital (LUMO), gives valuable insight into the electronic excitation states of a compound (Montanari et al., 2013). The HOMO is used to predict a molecule’s ability to donate electrons, while the LUMO predicts the molecule’s ability to accept electrons, both of which are important for charge transfer and chemical processes (Aloui et al., 2024a). The energy difference between the HOMO and LUMO (ΔE) is used to measure the relative stability and reactivity of a molecular species. The smaller the ΔE, the more reactive the molecule and the easier it is for electrons to move between the two (Abdulridha et al., 2020; Dege et al., 2022). To further explain the compounds studied, a range of quantum chemical descriptors was calculated (Aloui et al., 2024b), including ionization energy (I), electron affinity (A), electronegativity (χ), chemical hardness (η), chemical softness (S), electron chemical potential (μ), and the global electrophilicity index (ω). The obtained values are presented in Table 7 for comparison of the stability, electron distribution, and reactivity of the studied molecules. After examining AChE as a second target in the present study, the DFT analysis was carried out equally for compound D9, which has been derived from the most potent AChE inhibitor known so far (M7) to enable comparison between the electronic nature of the compounds with respect to DPPH and AChE activities.

**TABLE 7:** Calculated values of chemical reactivity parameters of M12, D8, and D9.

N	E_HOMO_	E_LUMO_	ΔE	MD	I	A	χ	S	ŋ	µ	ω
M12	−5.479	−0.993	4.486	1.58	5.479	0.993	3.236	1.122	2.243	−3.236	11.746
D8	−6.260	−2.095	4.165	2.29	6.260	2.095	4.178	1.041	2.082	−4.178	18.171
D9	−5.849	−1.890	3.959	6.43	5.849	1.890	3.869	0.990	1.979	−3.869	14.818

According to the study, compound D8 has a lower energy gap (4.165 eV) than Ref M12, suggesting higher chemical reactivity. A lower HOMO–LUMO energy gap, together with a favorable HOMO energy, facilitates electron donation to electron-deficient species such as the DPPH radical, thereby enhancing the predicted radical-scavenging (antioxidant) activity.

When comparing the compounds analyzed, compound D8 has the smallest energy gap relative to Ref M12; thus, it may be one of the strongest candidates for antioxidant activity. Similarly, compound D9 exhibited the lowest HOMO–LUMO energy gap (3.959 eV), supporting its enhanced electronic reactivity and its predicted potential as a promising AChE inhibitor.

**FIGURE 6:**
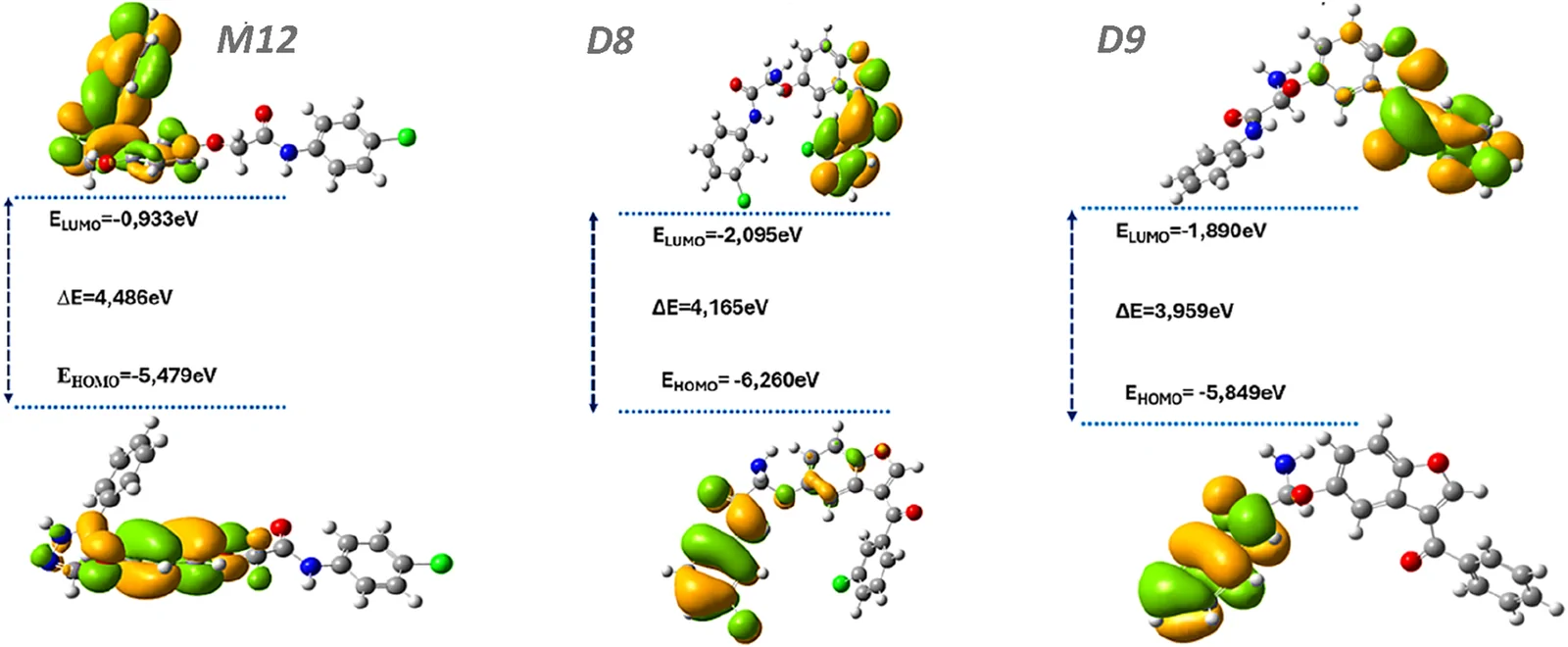
FMO representations of M12, D8, and D9 examined molecules. D9 molecule was previously reported by (El Fadili et al., 2025) under the Creative Commons Attribution License (CC BY).

The energy difference between a compound’s HOMO and LUMO orbital is generally proportional to bioactivity. When comparing the compounds analyzed, compound D8 has the smallest energy gap to Ref M12; thus, it may be one of the strongest candidates for an antioxidant. In addition, compound D8 demonstrates a higher Ionization potential (6.260 eV) and maximum electron affinity (2.095 eV); therefore, it has increased capacity to receive/donate electrons. In addition, the chemical hardness (η) or measure of molecular stability, correlating with the energy gap will be lower for compound D8, showing it reacts more readily and has potentially greater bioactivity than other tested compounds. Compound D8 also contains a higher chemical electron potential (μ) than ref M12, allowing it to react more frequently to gain and/or distribute electrons. Compound D8 also has a larger dipole moment (2.29 D) than ref M12 and indicates stronger interactions with adjacent molecules. In addition, due to compound D8 exhibiting a minimal energy gap, it can be classified as highly electronegative (4.178), confirming that it will exhibit a greater amount of reactivity. As illustrated in Figure 6, the compound’s electrophilicity index (ω) is higher than that of any of the other compounds, suggesting that this compound will transfer electrons from its HOMO to its LUMO at a higher probability than any of the other compounds.

Figure 6 shows Frontier orbitals from the molecules that were used in this study. The increased reactivity and predicted decreased stability of the two most reactive predicted molecules compared to our most reactive predicted compound (Reference M12) led to an investigation of the two compounds’ molecular electrostatic potential (MEP). The MEP can provide information regarding portions of each compound that can donate or accept electrons, which, in turn, helps to determine sites that are electrophilic or nucleophilic.

### 3.5 Molecular electrostatic potential analysis

**FIGURE 7:**
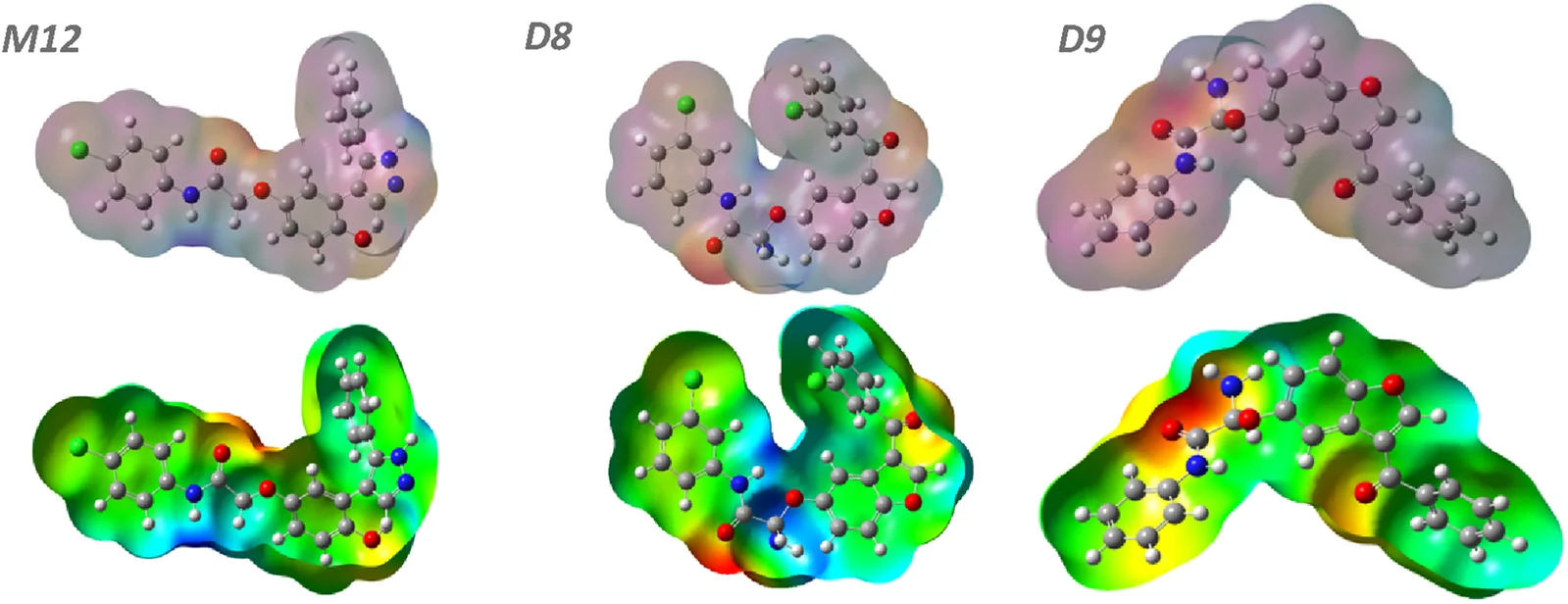
Molecular electrostatic potential of M12, D8, and D9 examined compounds. D9 molecule was previously reported by (El Fadili et al., 2025) under the Creative Commons Attribution License (CC BY).

Molecular Electrostatic Potential (MEP) map describes the charges in a molecule and thus allows for an evaluation of the interactions between two or more molecules. MEP analysis of compounds M12, D8, and D9 is depicted in Figure 7 and is important to biological recognition and hydrogen bonding, as well as for predicting both electrophilic and nucleophilic activity. The calculations of the molecular electrostatic potential (MEP) performed using the optimized geometry B3LYP 6-31G (d,p) allow for the analysis of electrostatic potential distribution and the prediction of reactive sites that may be targeted by electrophilic or nucleophilic species. Positively charged regions (shown in blue) correspond to electron-deficient regions susceptible to nucleophilic attack, while negatively charged regions (shown in red and yellow) indicate electron-rich regions susceptible to electrophilic attack. Figure 7 shows that compounds under investigation exhibit blue regions around the hydrogen atoms linked to nitrogen atoms, indicating low electron density and potential H-bond donor sites. In addition, their MEP maps reveal negative regions around the ketone group, suggesting substantial electron density and suitable places for hydrogen-bond acceptors and electrophilic attack. Compound D9 shows a distribution of electrostatic potential similar to the distributions of M12 and D8, having positive regions positioned around the hydrogens connected to the nitrogen atoms and negative regions positioned near carbonyl oxygen atoms. This shows that changes in the structure maintain the previous electrostatic properties of the series.

### 3.6 Molecular docking simulations

Molecular docking simulations were carried out for the most potent compounds against their intended biological targets. M12, the most potent synthesized compound, and D8, the most powerful designed antioxidant, were docked alongside the water-generating NAD(P)H oxidase (PDB ID: 2CDU), and D9, designed to be the lead compound in inhibiting acetylcholinesterase, was docked to AChE (PDB ID: 4EY7).

The docking results revealed that both M12 and D8 ligands exhibit strong affinities within the catalytic pocket, with binding free energies of −8.91 kcal mol for M12 and -9.22 kcal mol for D8. For M12, stabilization of the complex is mainly driven by multiple hydrogen bonds involving Met33, Glu32, and Thr9, complemented by Pi-Anion and unfavorable Donor-Donor contacts with Lys134 and Asn135 surrounding residues. The validated inhibitory effects of this compound might be due to the dual nature of its interactions (polar/hydrophilic) and its ability to create an attractive potential field. However, it is interesting to note that D8 had a more favorable binding score due to an increased number of stabilizing contacts formed between the various atoms of its structure than the other compounds. D8 forms one hydrogen bond with the side chain of Gly329, while simultaneously forming hydrophobic and π-alkyl contacts with the side chains of Ile44, Lys134, Lys331, and Val338. Although the existence of a Pi-sulfur repulsion interaction between Cys133 and the D8 structure was identified, this repulsion does not negate the overall strength of the entire interaction network between all of the D8 atoms, thus resulting in a stronger affinity of D8 than the other compounds tested within the same assay. Taken together, these findings suggest that while M12 effectively binds through hydrogen bond-driven interactions, D8 benefits from a synergistic combination of hydrogen bonding and hydrophobic stabilization, which may provide superior inhibitory potential.

**FIGURE 8:**
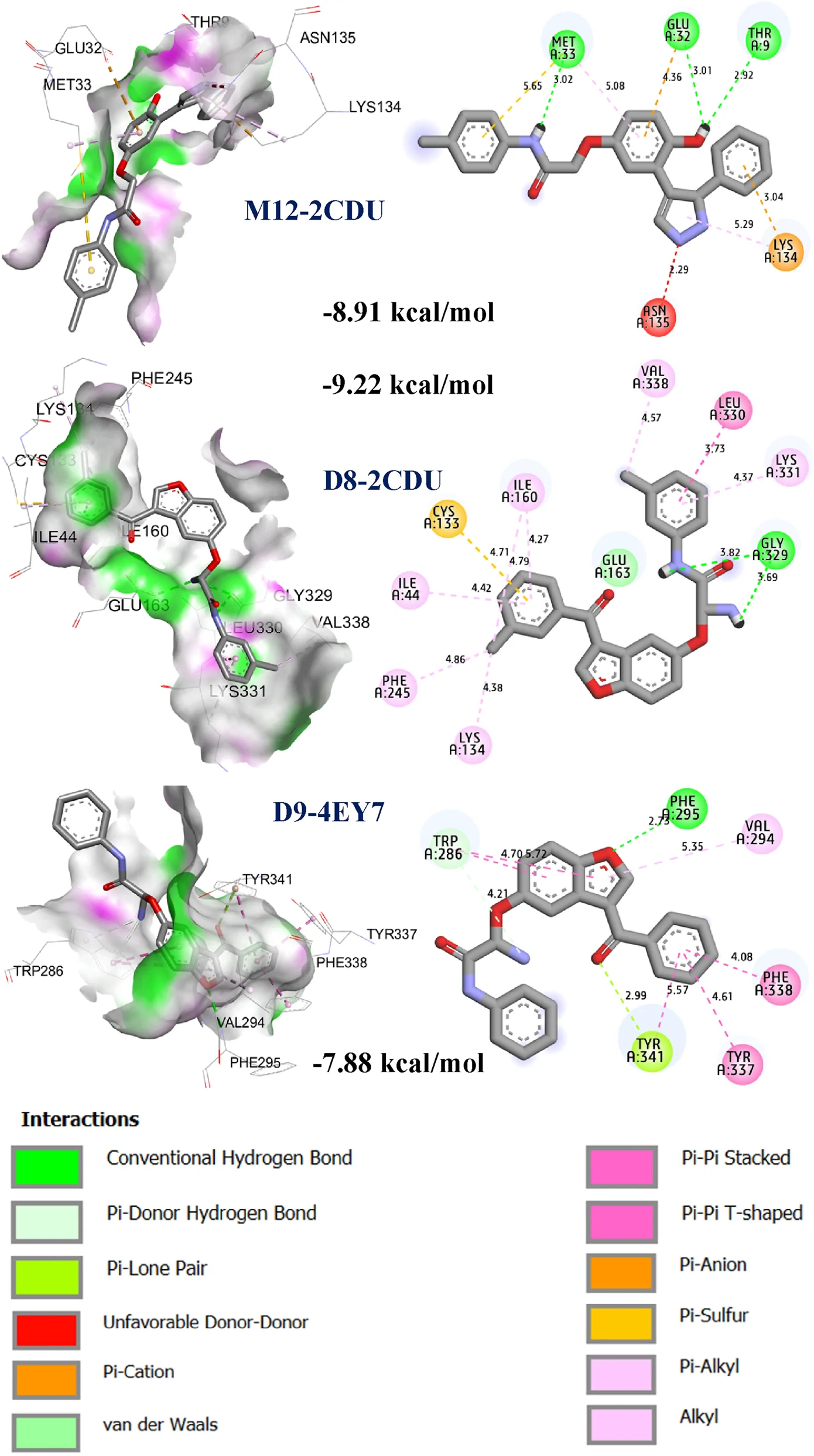
3D (right) and 2D (left) views of the inhibition mechanisms of (M12-2CDU) (D8-2CDU), and (D9-4EY7) complexes.

The new compound referred to as D9 was also docked to AChE (acetylcholinesterase, PDB ID: 4EY7), since it was specifically developed to act as an efficient AChE inhibitor. D9 displayed a binding affinity of −7.88 kcal/mol and was predicted to have a stable conformation in the active site. The complex was found to be stable thanks to the development of hydrogen bonds with Phe295 and Tyr341, and also π-π interactions between Trp286, Tyr337, and Phe338, in addition to π-alkyl interaction with Val294, supporting the advantageous binding position of D9 in complex with AChE enzyme, as presented in Figure 8.

#### 3.7 Redocking protocol

Redocking is a validation protocol for molecular docking simulation results. It aims to assess the reliability of the interactions obtained between the ligands and the target protein by verifying whether the candidate ligands are correctly positioned in the active sites of the protein. This step also consists of comparing the conformation of the cocrystallized (or native) ligand with that of the docked ligand under the same target preparation conditions, ensuring that the RMSD value in the superposition position remains below 2 Å, which indicates an acceptable accuracy of the docking process.

**FIGURE 9:**
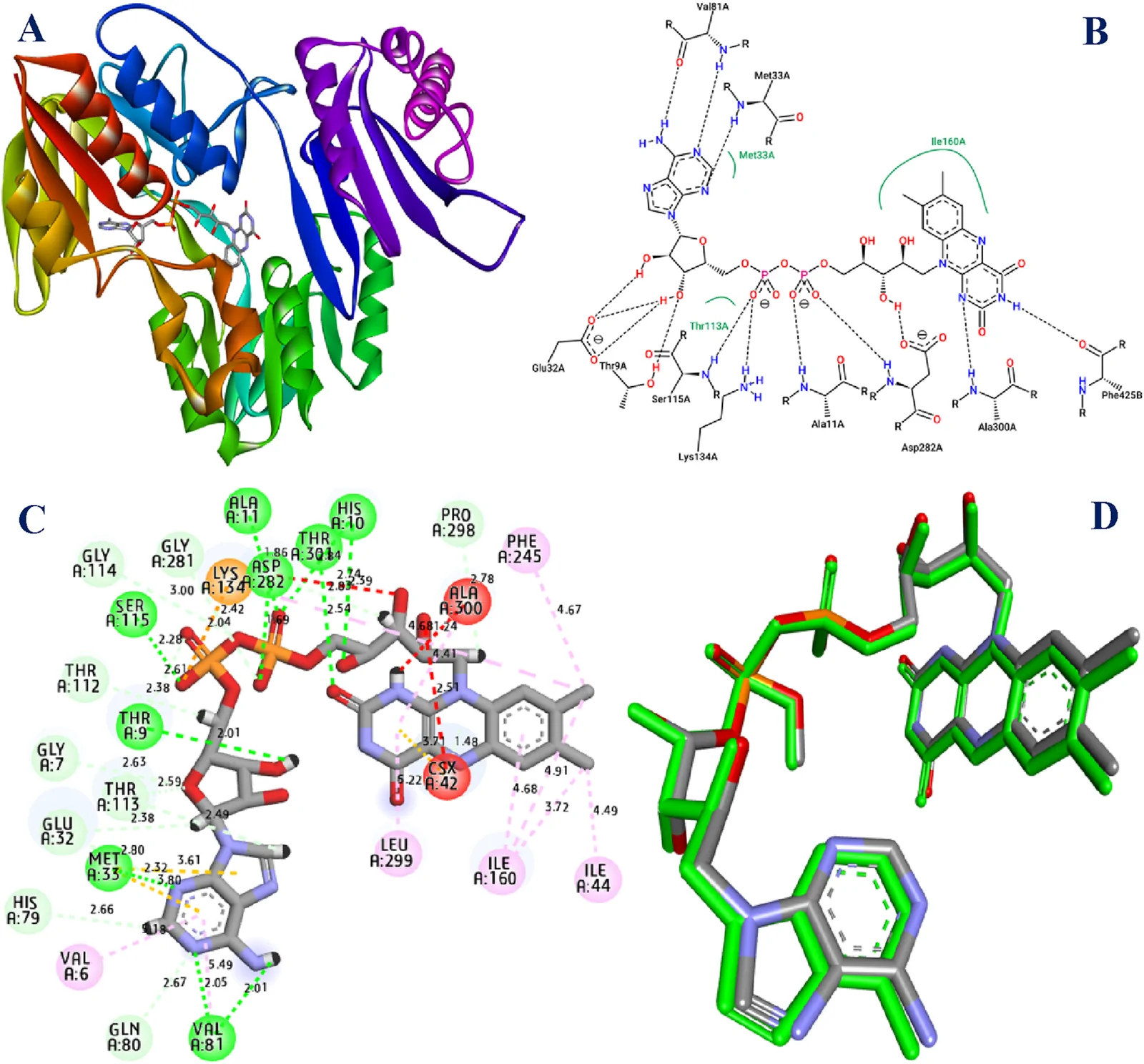
NADPH oxidase protein **(A)**, protein active sites **(B)** in complex with its native ligand, docked FAD ligand **(C)**, and superposition position with RMSD of 0.456 Å **(D)**.

First of all, Figure 9 clearly shows that Ala300, Asp282, Ala11, Lys134, Ser115, Thr9, Glu32, Val81, Met33 and Phe245 are the active sites of NADPH oxidase protein, which were detected towards flavin adenine dinucleotide (FAD), called ([[(2R,3S,4R,5R)-5-(6-aminopurine-9-yl)-3,4-dihydroxyoxolan-2-yl]methoxy-hydroxyphosphoryl] [(2R,3S,4S)-5-(7,8-dimethyl-2,4-dioxobenzo [g]pteridin-10-yl)-2,3,4-trihydroxypentyl] hydrogen phosphate). Nearly identical intermolecular contacts, such as Ala11, Asp282, Met33, Ala300, Val81, Ser115, Thr9, and Phe245, were obtained for the docked FAD ligand in complex with the 2CDU protein. In addition, M12 and D8 shared common and similar interactions, such as amino acid residues Met33, Thr9, Glu32, Phe245, and Lys134, confirming that both candidate molecules were successfully complexed not far from the 2CDU active sites. Second, the superposition of the co-crystallized FAD ligand onto the docked FAD ligand was achieved with an RMSD error of 0.456 Å without exceeding the 2 Å threshold, which confirms again that the docking protocol was successfully examined.

**FIGURE 10:**
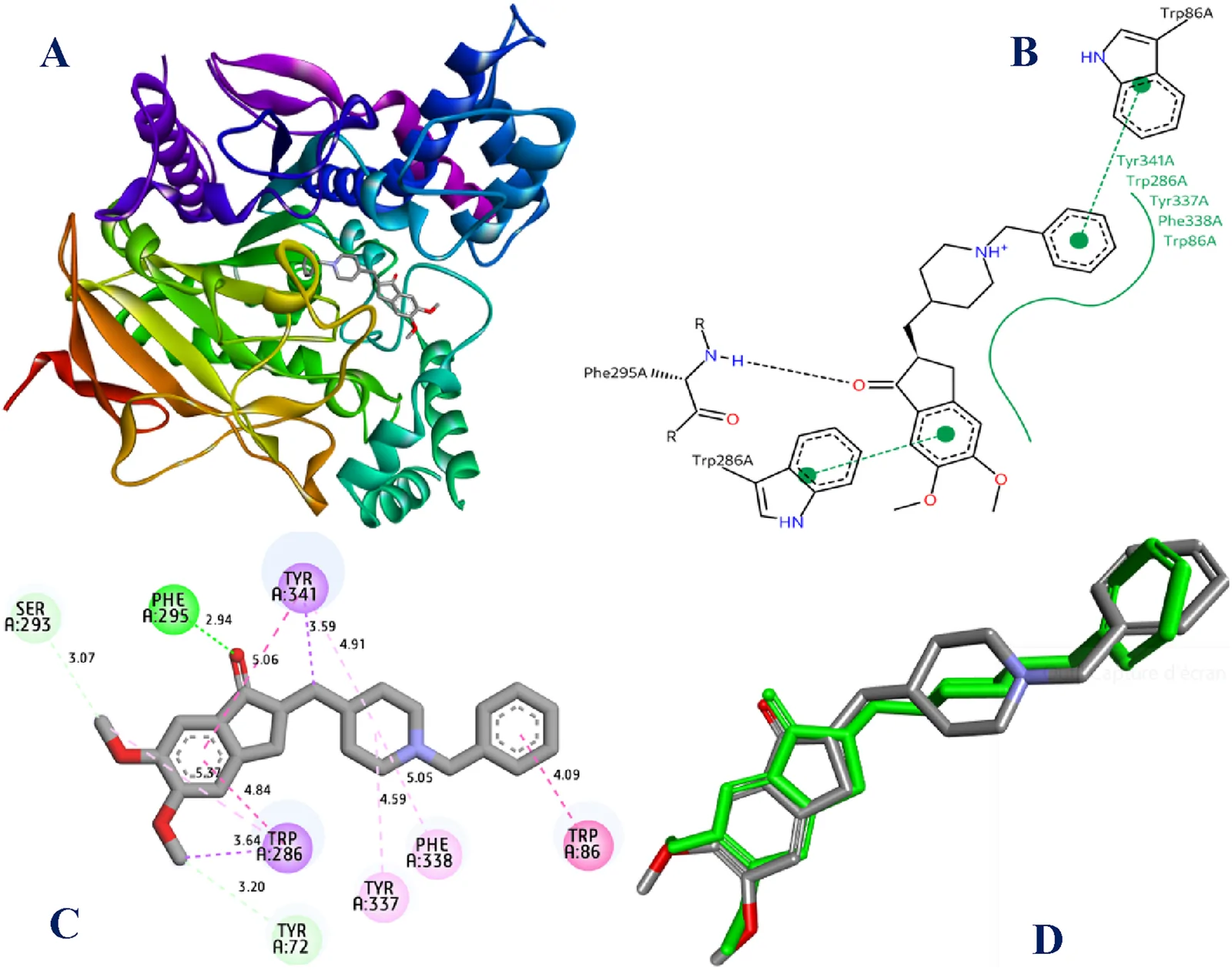
AChE cholinesterase protein **(A)**, protein active sites **(B)** in complex with its native ligand (Donepezil), docked Donepezil ligand **(C)**, and superposition position with RMSD of 1.523 Å **(D)**.

Similarly, the docking validation procedure for acetylcholinesterase (PDB ID: 4EY7) was evaluated through the redocking of the co-crystallized molecule Donepezil, as presented in Figure 10. The redocked position revealed the same molecular binding position as that of the crystal structure with an RMSD value of 1.523 Å, which is significantly lower than the generally accepted value of 2.0 Å, thus proving the correctness and validity of the docking procedure in predicting the binding of the molecule inside the AChE active site. Furthermore, the redocked Donepezil showed retention of key interactions with the catalytic-site residues as found in the crystalline complex. It is noteworthy that the designed molecule D9 has a similar interaction profile and established interactions with key residues like Trp286, Tyr337, Tyr341, Phe338, Phe295, and Val294. The observed similarity in the binding mode of D9 indicates that the molecule fits the AChE active site in the same pose as Donepezil, which has been recognized as a reference drug for AD and thus proves the validity of its rational design as a promising AChE inhibitor.

### 3.8 Molecular dynamics

To complement the docking analysis, 100 ns MD simulations were performed for the examined complexes. The RMSD and RMSF trajectories of the protein and ligands demonstrated that all three complexes reached equilibrium, confirming their structural stability over the simulation timeframe.

#### 3.8.1 Analysis of conformational changes with respect to RMSD

**FIGURE 11:**
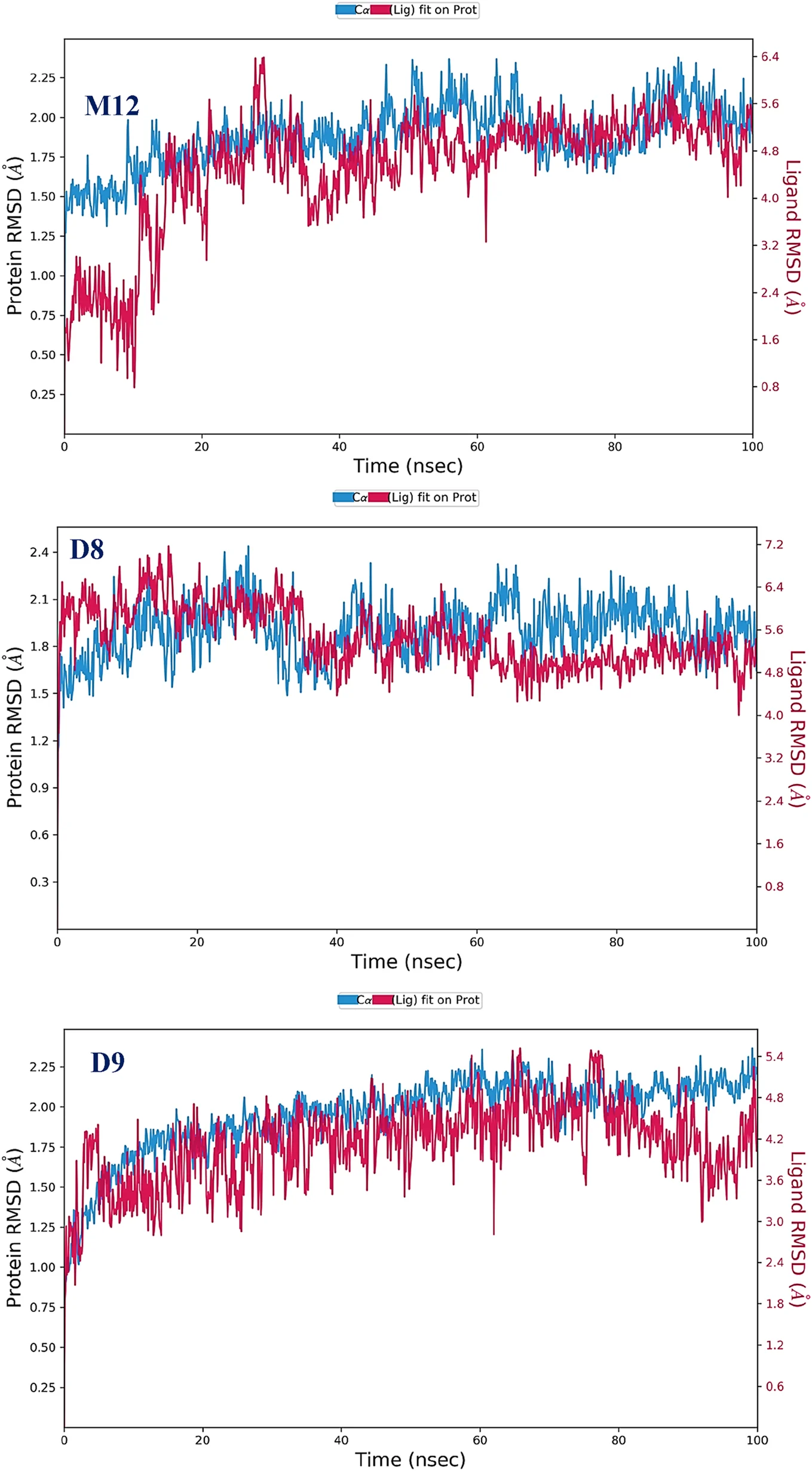
Conformational changes in RMSD values for M12, D8, and D9. D9 molecule was previously reported by (El Fadili et al., 2025) under the Creative Commons Attribution License (CC BY).

RMSD profiles presented in Figure 11 provide significantly different information regarding how the three protein-ligand systems were altered over their respective 100 ns MD simulations (Er-rahmani et al., 2024). While the receptor backbones maintained their overall structure, the three ligands, however, displayed significantly different dynamics within their respective binding sites. In the first 10 ns of simulation (0–10 ns), the backbones of the proteins quickly relaxed from their starting geometries and rapidly became stabilized at approximately 1.6–1.9 Å, representing natural equilibration of the loops and side chains in the solvated environment. After this equilibration, the structures of the proteins only slightly fluctuated from their original folds throughout the entire trajectory. The relative differences in RMSD for the ligands were far greater than the differences in the protein receptors. For ligand M12, the initial RMSD value was approximately 2.0 Å and increased during the equilibration phase before fluctuating between approximately 4.0 and 5.5 Å throughout the remainder of the simulation. The relatively large range and frequent spikes observed for ligand M12 in both the RMSD and RMSF plots indicate that ligand M12 underwent multiple conformational changes and/or rotations within the pocket and should be viewed as flexibly bound, while remaining associated with the binding site throughout the simulation. Unlike ligand M12, ligand D8 exhibited an initial RMSD of approximately 6.0 Å, followed by moderate fluctuations between approximately 4.5 and 6.0 Å during the 100 ns simulation. These fluctuations suggest that D8 underwent conformational adjustments while maintaining a stable binding orientation within the active site.

Similarly, the (D9-4EY7) complex experienced a 100-nanosecond MD simulation to check its dynamic stability. The AChE backbone achieved equilibrium within the first 15–20 nanoseconds and then remained stable, with RMSD values fluctuating around 1.8–2.2 Å, showing the continuity of the protein structure during the simulation. On the other hand, the D9 ligand showed RMSD values in a range of approximately 3.2–5.2 Å. The ligand RMSD is calculated after the fitting of the protein, indicating changes that mainly demonstrate conformational adjustments of the ligand within the AChE binding site. Most importantly, no sharp or continuous increase in the ligand RMSD was observed, showing that D9 was still in position in the binding site and was trying to adapt to the most favorable conformation.

In summary, while all three systems, M12, D8, and D9, have been found to be stably attached throughout their 100-nanosecond simulations, they behaved differently in terms of conformational behavior. The M12 ligand demonstrated relatively high flexibility as indicated by larger RMSD deviations, reflecting higher conformational freedom in the binding site. On the contrary, the D8 receptor experienced smaller RMSD changes after stabilization, which points to the relative rigidity of D8 orientation. The conformational profile of the D9-AChE complex also remained stable throughout the simulation, where the protein as well as the ligand were stable after equilibration and showed only minor fluctuations throughout the simulations.

#### 3.8.2 RMSF-Based conformational dynamics

**FIGURE 12:**
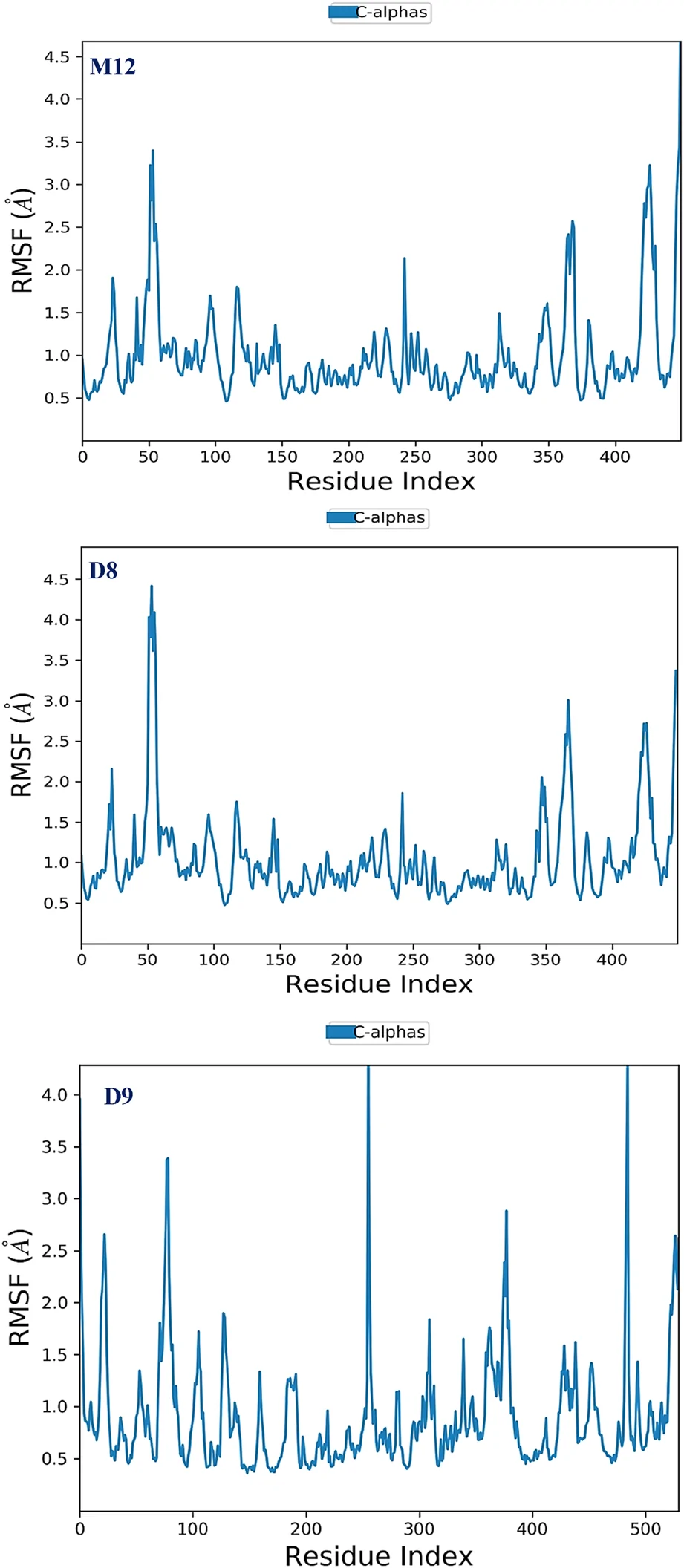
Conformational changes in RMSF values for M12, D8, and D9. D9 molecule was previously reported by (El Fadili et al., 2025) under the Creative Commons Attribution License (CC BY).

Results from root mean square fluctuation (RMSF) analyses of the flexibility of the protein over time (100 ns) in complex with M12, D8, and D9 separately are shown in Figure 12. The three complexes show expected patterns for fluctuation: greater flexibility at endpoints and loops than at the core; however, the differences between the two complexes are in areas surrounding the binding site. For the M12-ligand complex, the majority of residues (that is, the vast majority of protein backbone) demonstrated fluctuations in the range of 0.5–1.5 Å. For this complex, large peaks were observed in the range of ∼50, 250 and 400 AA, in these regions RMSF values reach approximately 2.5 Å and in the most flexible region (near residue 400 between ∼3.0-3.3 Å). These residues represent the expected flexibility of solvent-exposed loops and terminal residues, which always demonstrate some form of increased mobility relative to the core of the protein. The generally low level fluctuations across most of the molecule indicate that the M12 does not alter significantly the overall stable, stiff structure of the receptor scaffold, and some evidence for flexibility at residue 400 indicates some level of localized structural adaptability around the binding site. Similar results were obtained in the D8-liganded receptor; the global pattern is similar, however, there are areas of increased flexibility in certain regions (i.e., more than in M12). The highest level of flexibility for this complex occurred at ∼50 AA; however, the RMSF value was greater than 3.0 Å (which is greater than in the M12 complex).

Fluctuations are observed at moderate levels for ∼230 Å and ∼350 – 400 Å, indicating there is an increase in the movement of loops after D8 binds; therefore, after D8 binding to the protein, residuals that are near or less than 1.5 Å continue to indicate that the protein fold is stable. The overall larger fluctuation peak occurring near the N-terminus potentially indicates that ligand binding induces local conformational breathing that creates space within the binding site for the ligand to fit. The RMSF profile of the (D9-4EY7) complex revealed low levels of fluctuation for most residues of AChE. The average value of RMSF falls between 0.4 and 1.0 Å, which implies that the protein architecture is relatively stable during the course of the 100 ns simulation. High RMSF values could only be related to a limited number of residues at flexible loops and terminal parts, with the largest peak being in the neighborhood of 4.3 Å. The given changes are characteristic of structurally flexible regions, and they did not cover bigger regions of the protein structure. In addition, the level of flexibility for the residues of the AChE binding site is also low because no significant changes in the structure of D9 occurred while being inside the active site. In terms of the global stability of the protein, it appears that all three complexes are able to maintain global stability, but they display different fluctuation distributions. M12 produces moderate to ample fluctuation over a few residues (a smaller distribution of fluctuations), while D8 results in larger (but modally concentrated) local fluctuations that are found in the N-terminal loop. It would follow then that binding D8 to the protein may cause a compounding of conformational flexibility to stabilize its binding orientation within the active site through induced conformational breathing for the loop regions of the protein. The pattern of RMSF values observed would be in agreement with those observed for RMSD values in terms of how the examined ligands interact with their respective protein targets (M12 having lower RMSF and RMSD, while D8 has a higher RMSF with a much lower RMSD). M12 is mostly stable with binding to the protein, while D8 has demonstrated an overall goal of reaching a stable binding position, and all of the areas that require localized flexibility insertion into the binding site were already pre-defined before binding D8. Similarly, Moreover, the RMSF profile of the D9-4EY7 complex confirmed its RMSD characteristics, which showed that flexibility was mostly restricted to the loop and terminal segments of the complex while the AChE binding pocket showed stability all throughout the simulation. This further substantiates that D9 retains its position within the active sites during the 100 ns of the simulation.

##### 3.8.3 Protein-ligand interactions

**FIGURE 13:**
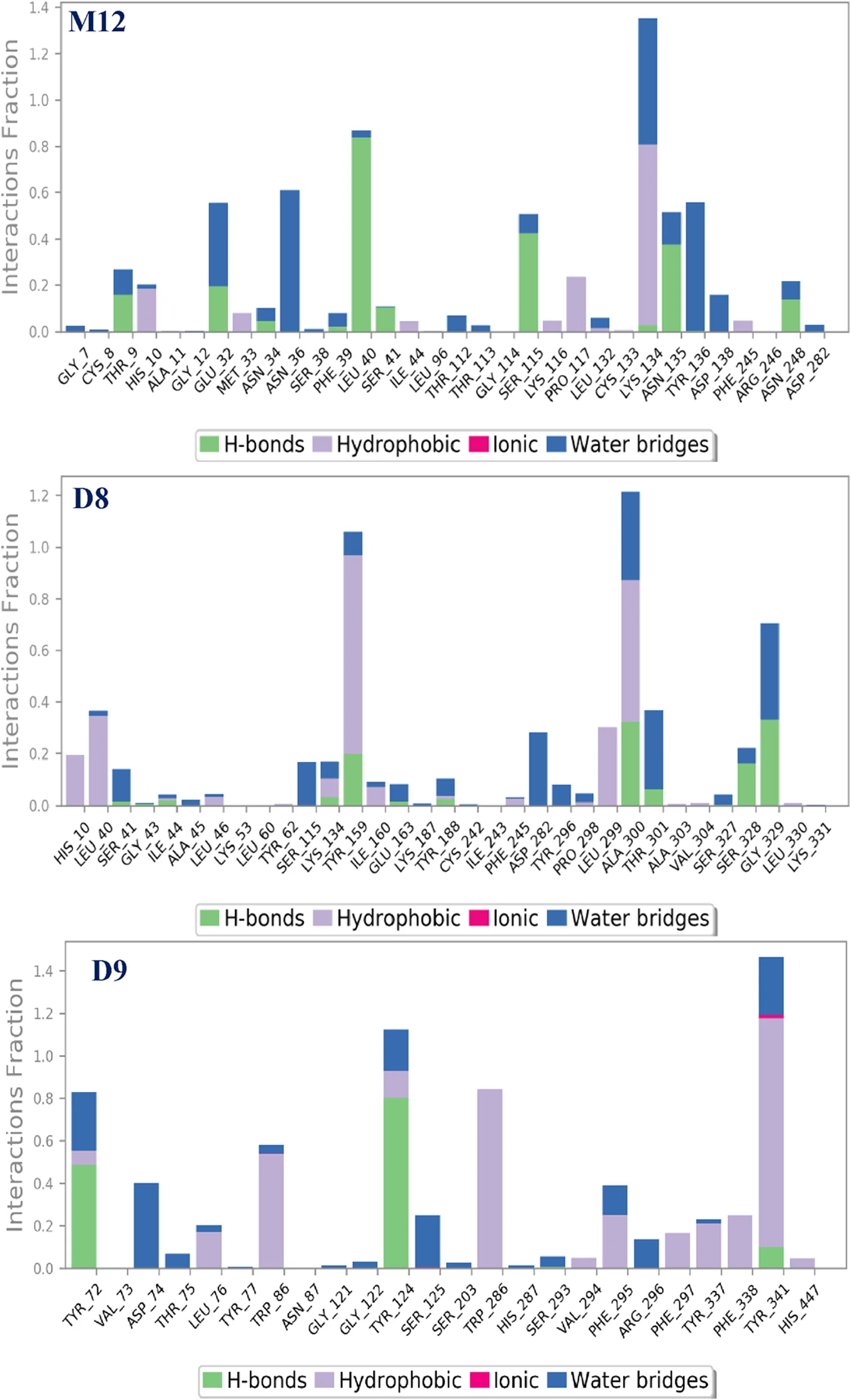
Results from protein-ligand interactions for M12, D8, and D9. D9 molecule was previously reported by (El Fadili et al., 2025) under the Creative Commons Attribution License (CC BY).

The binding of M12, D8, and D9 to their respective complexes is characterized by different patterns of interaction as illustrated in Figure 13. Multiple side chain interactions stabilize the M12 complex with substantial contributions from ASN134 and ASN136 through hydrogen bonding and water-mediated contact. The other stabilizing contributions come from SER40, ASN36, LYS206 and the hydrophobic contributions of PHE41 and ILE44, showing that M12 is stabilized by an extensive, flexible network that is predominantly composed of polar and water-bridge interactions. The D8 complex has a more limited but localized binding profile. The hydrophobic residues TYR134 and ALA299 provide continuous hydrophobic and water-mediated interactions, while the hydrogen bonds between SER65, THR209 and GLU230 represent further stabilizing components. The greater predominance of hydrophobic interactions, supplemented by selective hydrogen bonds, indicates a more compact and uniform binding mode than that of M12. Similarly, the D9-4EY7 complex showed a stable interaction profile throughout the 100 ns simulation. The complex was mainly stabilized by persistent hydrogen bonds with Tyr72 and Tyr123, in addition to extensive hydrophobic interactions with Tyr77, Trp203, Phe294, Phe296, Tyr337, Phe338, and Tyr341. Moreover, water-mediated interactions with Tyr72, Val73, Tyr123, Ser124, Phe294, and Arg295 helped enhance the stability of the complex. The pattern of interactions indicates that D9 was consistently hosted in the AChE binding pocket during the simulation, which complies with RMSD and RMSF results.

In summary, M12 displays an array of water-assisted interactions showing a flexible binding mechanism; however, D8 relies on fewer but stronger hydrophobic and bridging interactions, which are consistent with its greater stability indicated by RMSD and RMSF analyses. Likewise, D9 displayed a balanced interaction network consisting of stable hydrogen bonds, hydrophobic interactions, and water bridges, showing a stable binding mode over the simulation. The results obtained further confirm the docking results and reinforce the ability of D9 to serve as a potential acetylcholinesterase inhibitor.

##### 3.8.4 Protein-ligand contact interaction timelines

**FIGURE 14:**
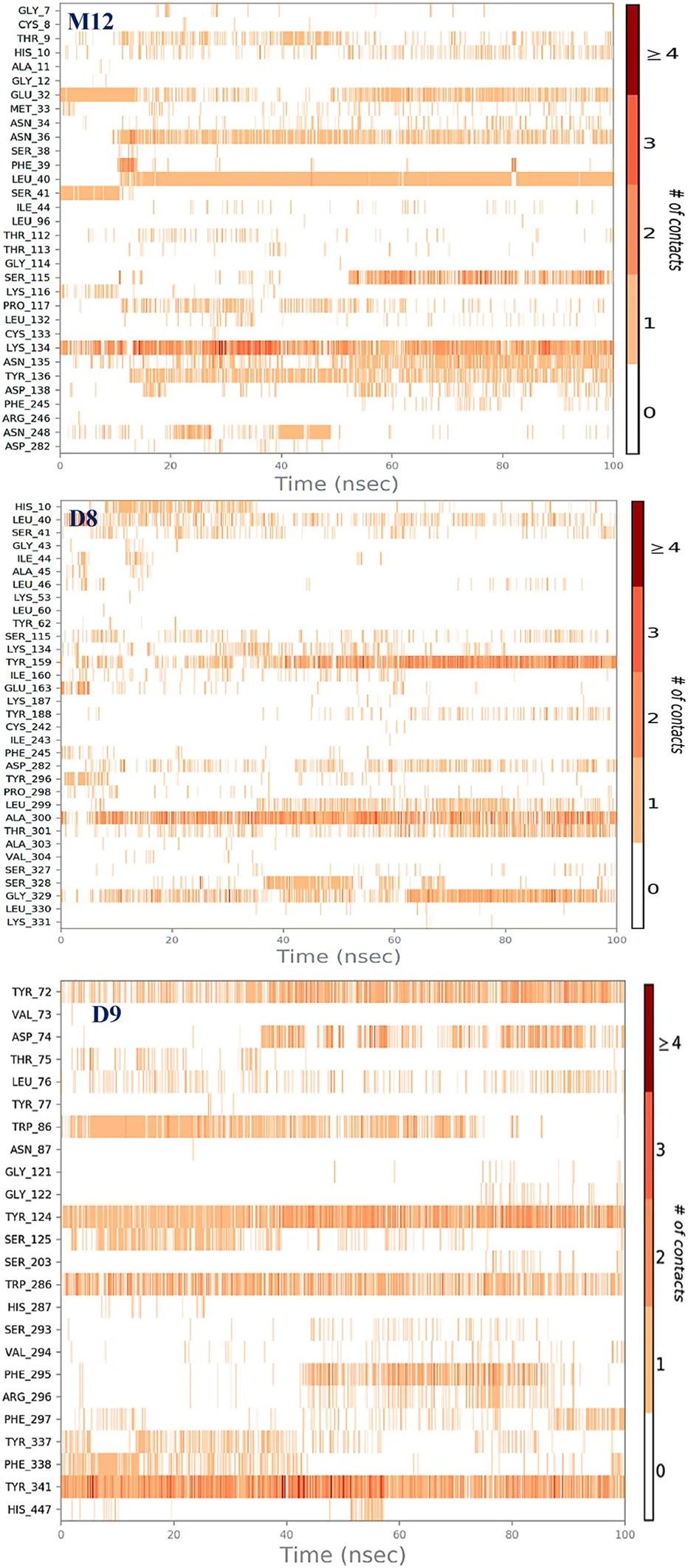
Time-Resolved Protein-Ligand contact patterns of M12, D8 and D9.

The use of interaction timelines allows the evaluation of the comparative stability of ligand binding, M12, D8, and D9, to the binding site of the respective target proteins, as well as the amount of time each ligand remains in contact with its corresponding active site amino acid residues through the entire 100 ns molecular dynamics simulation, as demonstrated in Figure 14. Throughout the simulation, M12 establishes both consistent and repeatable binding contacts to multiple residues in the binding site, specifically Leu40 and Lys134. Hydrophobic (hydrophobic residue to hydrophobic residue) and hydrogen-bonding (hydrogen bond between an aromatic) types of interactions dominate M12’s binding characteristics and provide a stable binding profile. Additionally, the repetitive interactions of Lys134 as well as those with polar residues such as Ser115 demonstrate the critical role of electrostatic/hydrogen bonding in preventing dissociation of M12 from the NADPH oxidase target. M12’s overall profile suggests that M12 can form a relatively stable mode of binding to NADPH oxidase despite fluctuations that occur between certain pairing interactions. In contrast, D8 formed a wider range of contacts, with several of the main contacting residues being Ala300, Tyr159, and Leu40. Contacting residues can be found between two residues for an extended number of simulations (e.g., Leu299 and Ala300), providing D8 with favourable stabilisation through hydrophobic interactions. Furthermore, the continued interaction of D8 with polar residues (e.g., Ser115, Glu113, and Tyr159) will provide additional hydrogen-bonding support to D8’s overall stability. Similarly, the D9-4EY7 complex demonstrated stable protein-ligand interactions throughout the entire 100 ns duration of the MD simulation. The timeline of these interactions revealed the continuous presence of contacts with Tyr72, Tyr124, Trp286, and Tyr341, suggesting the key contributions of these residues to the stability of D9 in the AChE binding pocket. Other residues, including Phe295, Tyr337, and Phe338, maintained their contacts during a significant part of the simulation, while the remaining residues, including Asp74, Ser125, Ser293, Val294, Arg296, and Phe297, contributed only with limited interactions. The overall conclusions indicate that D9 was firmly established in the AChE active site, confirming the stability of the complex reflected in the RMSD and RMSF analyses.

In comparison to M12, the binding interaction of D8 has a larger variety of representatives (or interacting groups) across the entire binding site region. This indicates that D8 assumes a relatively longer binding conformation than what M12 is expected to do; therefore, D8 will utilize a combination of both hydrophobic and polar interactions in order to create an overall more stable complex. Both ligands show a prolonged period of binding; however, M12 binds to its target using a narrower range of relatively similar contact areas than does D8. The significant difference in the use of contact areas may account for the increased stability of D8 as noted throughout this study’s simulated experiments. Additionally, the continuous interaction pattern exhibited by D9, especially the regular interactions with Tyr72, Tyr124, Trp286, and Tyr341, affirms the stable presence of the designed compound in the active site of AChE during the whole simulation of 100 ns, supporting its prospect as a competitive acetylcholinesterase inhibitor.

#### 3.8.5 Comparative analysis

For greater reliability, the molecular dynamics results of M12, D8 and D9 were compared with those of referenced drugs: Donepezil as known AD drug and flavin adenine dinucleotide (FAD) native ligand co-crystallized in the NADPH oxidase protein, which were also subjected to the same experimental conditions simulated during 100 ns of molecular dynamics time.

**FIGURE 15:**
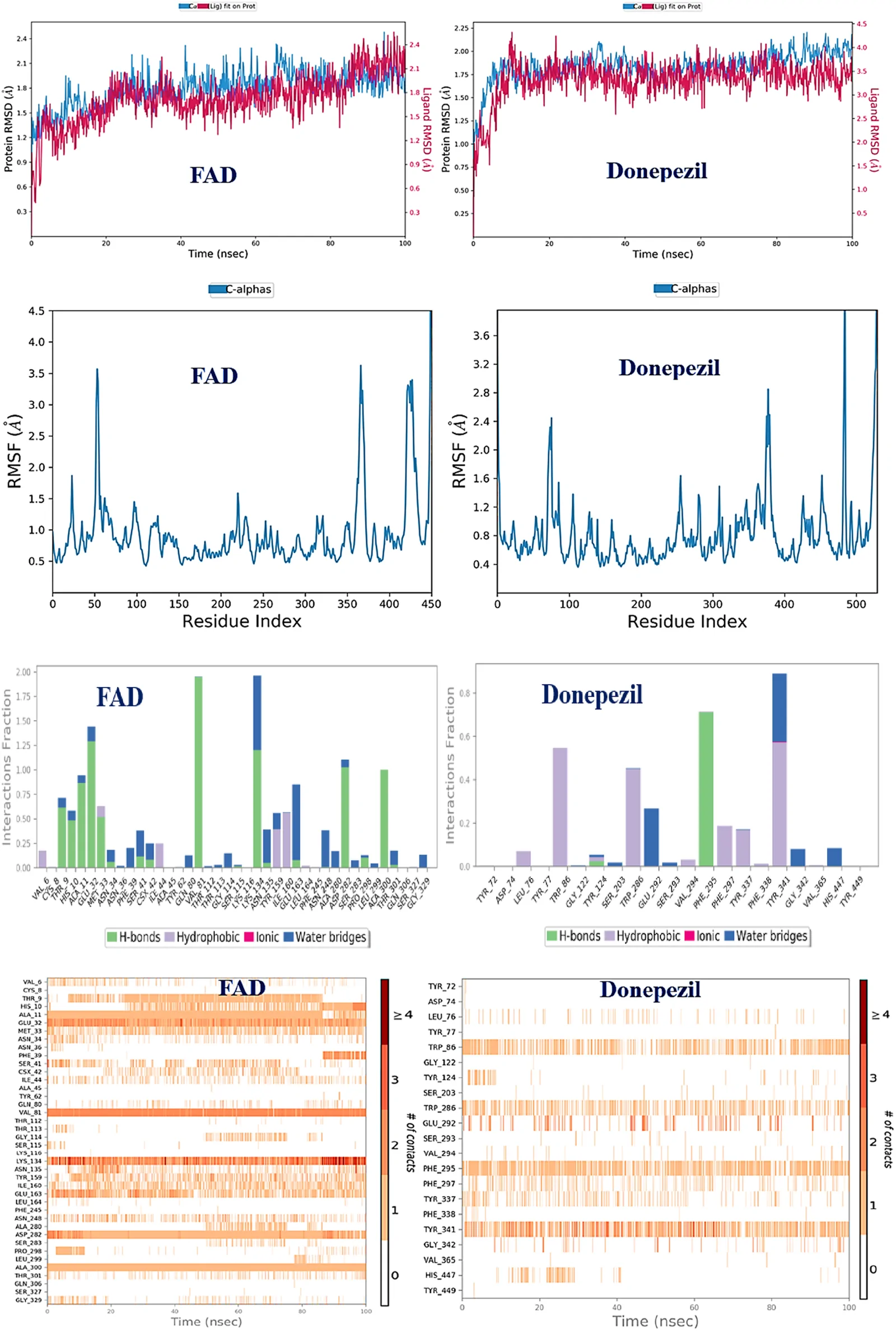
Dynamics results of RMSD, RMSF, and intermolecular contacts in complex with NADPH oxidase protein.

The results obtained in Figure 15 revealed an excellent level of thermodynamic stability of both native and referenced ligands, justified by minimal conformational changes in RMSD and RMSF values, and the strong contribution of the receptor active sites to achieving this level of molecular stability, especially Val81, Glu32, Asp282, and Lys134, by creating more rigid interactions very similar to those obtained for candidate compounds M12 and D8.

Similarly, the donepezil-AChE complex (PDB ID: 4EY7) displayed good stability during the molecular dynamics simulation of 100 ns, with a quick stabilization of the RMSD of the protein backbone of around 1.7–2.1 Å while the RMSD of the ligand fluctuated around 3.0–3.8 Å, implying that donepezil stayed bound in the active sites though conformational changes were observed. The RMSF analysis also showed that most of the residues of AChE exhibited changes below 1.0 Å, with most of the flexibility shown in the terminal loops rather than in the catalytic pocket. The interaction analysis confirmed that stable interactions were maintained between donepezil and important residues of the active site, namely, Trp86, Trp286, Phe295, Phe297, Tyr337, Tyr341, and His447. Out of these residues, Tyr341, Phe295, and Trp86 showed the greatest occupancy of interaction throughout the simulation, which supports the idea that hydrophobic and π–π stacking are the decisive forces for stabilization of the donepezil-AChE complex. In the case of the AChE target, the D9-AChE complex showed similar dynamics as the reference donepezil–AChE complex. Both models demonstrated that the protein backbones remained stable throughout the 100 ns simulation and maintained stable interactions with critical residues of the catalytic active site, confirming that D9 is a stable ligand. Also, for the NADPH oxidase target (2CDU), both M12 and D8 showed stability profiles similar to those of the native FAD complex, with low fluctuations and good interactions with active-site residues, indicating the reliability of the designed compounds as potential NADPH oxidase inhibitors.

Recent computational studies reported that apocynin, when docked into the NADPH-dependent oxidase model (PDB ID: 2CDU), exhibited a binding energy of approximately −8.3 kcal mol^-1^, forming hydrogen bonds with Ser115A and Thr9A active sites, located near the flavin-binding region of the enzyme (Mhya et al., 2023). In contrast, most available docking studies on apocynin and VAS2870 have focused on human NOX isoforms and their subunits, rather than on the bacterial NADPH oxidase analogue represented by 2CDU. The native FAD ligand was also bound in the same model, revealing interaction features consistent with those reported by Santos, W. H. dos, et al., further strengthening the suitability of 2CDU for comparative docking analyses (Santos et al., 2021).

## 4 Conclusion

In this study, a 3D-QSAR strategy was applied to guide the rational design of novel pyrazole- and benzofuran-based derivatives as potential anti-Alzheimer’s agents with anticholinesterase and antioxidant properties. The resulting predictive models offered critical structural insights that informed the design of new compounds with enhanced activities. DFT calculations were used to analyze the electronic properties, and ADMET predictions were used to estimate pharmacokinetics and toxicity, which are generally accepted as characteristics of drug-like molecules. Molecular docking studies showed the presence of very strong and specific binding interactions between AChE and NADPH oxidase binding sites towards D8 and D9 designed compounds; molecular dynamics simulations confirmed that those candidate complexes are thermodynamically stable under physiological conditions. Overall, this combined computational approach has identified D8 and D9 as lead candidates due to their superior binding stability compared to the reference ligands (FAD and Donepezil) and their acceptable pharmacologic attributes, thereby warranting experimental validation as promising therapeutic agents for the treatment of Alzheimer’s disease.

## Data Availability

The original contributions presented in this study are included in the present article. Further inquiries can be directed to the corresponding author(s).
